# Improving the value of population health data for health policy and decision-making using machine learning algorithms in EQ-5D-5L index estimation

**DOI:** 10.1038/s41598-025-32123-6

**Published:** 2026-01-30

**Authors:** Áron Hölgyesi, Zsombor Zrubka, Mehdi Neshat, Viktor Jáger, Áron Kincses, Levente Kovács, László Gulácsi, Seyedali Mirjalili, Márta Péntek

**Affiliations:** 1 Health Economics Research Center, University Research and Innovation Center, Óbuda University, 96/B Bécsi út, Budapest, 1034 Hungary; 2 Innovation Management Doctoral School, Obuda University, Budapest, Hungary; 3 Center for Artificial Intelligence Research and Optimization, Torrens University Australia, Brisbane, Australia; 4 Hungarian Central Statistical Office, Budapest, Hungary; 5 Institute of World and Regional Economics, University of Miskolc, Miskolc, Hungary; 6 Physiological Controls Research Center, University Research and Innovation Center, Obuda University, Budapest, Hungary; 7 University Research and Innovation Center, Obuda University, Budapest, Hungary

**Keywords:** Minimum European Health Module, EQ-5D-5L, General population, Machine learning, Artificial intelligence, Quality of life, Machine learning, Outcomes research

## Abstract

This study aimed to estimate patient-level EQ-5D-5L index scores using routinely collected sociodemographic and Minimum European Health Module (MEHM) data from seven extensive population surveys (*N* = 9,324). Fourteen machine learning (ML) models were compared in five research scenarios using the recently developed G score. Based on the performance ranking shown across scenarios, AdaBoost emerged as the best model (mean rank 2.87), followed by Multilayer Perceptron (MLP) and XGBoost (mean ranks 2.94 and 3.60, respectively). AdaBoost achieved the best results when no imputation was done and both sociodemographic and MEHM data were included (G = 0.955), but its performance declined when the estimation was based solely on sociodemographics (G = 0.871). The results confirm that the EQ-5D-5L index can be well predicted from existing statistical data using ML methods and that the MEHM improves the estimation. Our findings also highlight the potentially undesirable effects of data imputation in ML-based estimations. The methods presented in this study enhance the usability of existing health data, giving analysts and decision-makers a practical way to populate health-economic evaluations when primary data collection is impractical or impossible. Nonetheless, even advanced ML algorithms have limitations, so direct EQ-5D-5L data collection should remain the preferred approach whenever feasible.

**Supplementary Information:**

The online version contains supplementary material available at 10.1038/s41598-025-32123-6.

## Introduction

 Over the past decades, healthy life expectancy has been advancing slower than life expectancy, indicating a global expansion of ill health^1^. People live longer and may spend even decades with chronic conditions and disabilities. Hence, besides increasing life expectancy, improving the quality of the life years gained has become a key policy target in many countries. Combining impairment due to diseases (morbidity) and the length of life (mortality) in a single figure, summary measures of population health, such as the quality-adjusted life years (QALY), have been adopted to monitor the health status of a population, health inequities, social acceptability of different health states, and effects of policies and interventions^2–4^.

As healthcare systems are moving towards patient-centered care, patient-reported outcome measures (PROMs) have become the widely accepted instruments for health assessments^5^. PROMs can be categorised as non–preference-based (nPROMs) and preference-based (pPROMs) tools. Both nPROMs and pPROMs measure self-perceived health of individuals but pPROMs also attach preference weights, the so-called utility values or utilities, to different health states^6^. In other words, pPROM utility values reflect societal preferences regarding how good or bad a health state is, expressed in a single index score. These utility values are used to compute QALYs for health economic analyses and resource allocation decisions^7,8^. The most widely used pPROM instrument to calculate QALYs is the EQ-5D health-related quality of life instrument, developed in 1990 ^9^. Although the EQ-5D is increasingly used in clinical trials, patient registries and population studies, directly measured data are often lacking; therefore, EQ-5D utility values for health economic evaluations are often derived via mapping algorithms from nPROMS, which are ubiquitous in clinical research^10^.

In previous studies, numerous nPROMs have been used as source measures, although general instruments have received less attention compared to condition-specific ones^11^. Yet, general instruments are particularly well-suited for assessing population-level health status and are thus more appropriate for estimating pPROMs in contexts where the primary goal is their use in public health or health policy studies, or the health economic evaluation of system-wide interventions^8,12^. The Minimum European Health Module (MEHM) represents an ideal candidate for these purposes, as it is a standardized instrument developed by Eurostat, regularly administered in European statistical and social surveys since 2002^13^. It captures health status through three items (self-perceived health, chronic morbidity, and activity limitation) that can serve as strong predictors for estimating missing EQ-5D utility values in large, diverse real-world datasets. While MEHM data are widely available and consistently collected, EQ-5D data (although commonly used in clinical trials and registries) are rarely included in routine health or population surveys. Despite its clear potential, the MEHM has remained relatively underutilized in EQ-5D estimation studies, highlighting the need for research that systematically evaluates its applicability and provides guidance on suitable methods for its inclusion^14^.

In addition to the quantity and quality of the input data used for estimation, the statistical approach applied during mapping also plays a critical role. In recent years, machine learning (ML) methods have been introduced in pPROM utility mapping studies^15–18^. Compared to conventional mapping techniques (typically based on regression models) ML approaches offer several advantages: they are better equipped to capture complex relationships between variables and are more suitable for handling non-linear interactions^19^. These properties make ML particularly advantageous when analyzing large, high-dimensional datasets. Therefore, as the healthcare sector also faces the rise of big data, the relevance of ML-based methods continues to grow. A vast amount of real-world data (RWD) is being generated in digitalized health systems, and with the increasing adoption of wearable devices, this data accumulation is expected to even further increase, creating a rich and inexhaustible source of information^20–22^. However, these data are often unstructured, fragmented, and incomplete, raising important methodological challenges beyond the estimation itself, such as what imputation strategies can be implemented effectively in ML studies^23,24^. The mapping of health state utilities from such diverse datasets requires a fundamentally different approach, aimed at maximizing the utility of existing information and supporting health decision-making with the best available evidence^21^.

Accordingly, previous studies have reported that machine learning models can, in certain cases, outperform classical mapping approaches, although the findings remain controversial. When predicting EQ-5D utility values from a mixed set of demographic and health-related variables, ML-based and classical regression methods have shown comparable overall performance^14^. Yet, the superiority of one approach over the other largely depended on the target population, the set of input variables, and the metrics used to assess model performance. A recent large-scale systematic review also found that ML models generally outperformed classical regression methods by an average of only 6.2% in terms of prediction error and explanatory power^11^. However, their performance varied greatly depending on the specific task and the evaluation metric used. It has been also described that while ML methods predicted utilities more accurately than regression-based models, algorithmic bias was still observable, particularly in poorer health states^14,17,18^. These findings clearly demonstrate the potential of ML in health utility mapping, but offer only limited guidance for researchers in selecting the most appropriate models for their specific purposes. A major limitation of currently available studies is that they typically assess only a small number of ML algorithms and rely on a narrow set of performance metrics^11^. This makes it difficult to systematically compare models and draw robust conclusions about their relative performance, underlining the need for further research that provides comprehensive insights into the applicability of diverse ML methods across various contexts.

In the present study, we aimed to address these research gaps by exploring how patient-level EQ-5D-5L utility values can be estimated using routinely collected and widely available sociodemographic and MEHM data through advanced machine learning–based mapping techniques. Our objective was also to conduct a comprehensive evaluation of multiple ML models using a broad set of performance metrics to identify which methods are best suited to generate sufficiently accurate, fair, and unbiased predictions from the available data. Additionally, we examined the impact of various multiple imputation strategies for handling missing data on prediction accuracy.

## Results

### Descriptive analyses

#### The database

After merging the 7 studies, the full database consisted of twelve variables (sex, age, education, settlement type, marital status, employment, number of household members, income, EQ-5D-5L index value and the three items of the Minimum European Health Module - MEHM) and 9,324 observations. The average proportion of missing data in the database was 3%, ranging from 0% to 22% per variable. (Supplementary Table 1.)

After stratified random splitting, the training, validation and test datasets contained 5,595, 1,871 and 1,858 observations, respectively. When deleting observations with missing values (Scenario 1 and 2), the number of remaining observations in the training, validation and test datasets were 3,699, 1,196, and 1,253, respectively. After performing 30 multiple imputations (Scenarios 3–8), the number of observations increased to 167,850 in the training, 56,130 in the validation, and 55,740 in the test dataset.

In all datasets, the total number of variables was nine (the EQ-5D-5L index plus the eight demographic variables) in Scenarios 1, 3, 5 and 7, while there were twelve variables (the EQ-5D-5L index plus the eight demographic variables and the three questions of the MEHM) in Scenarios 2, 4, 6 and 8.

#### Respondent characteristics

The characteristics of respondents in the training, validation and test datasets by key demographic variables and the EQ-5D-5L index are shown in Table 1. The distribution by sex was similar between the general population and all samples. However, in terms of age, education and type of residence, the samples showed the characteristics typical of online samples: respondents in the youngest and oldest age groups, those with primary education and those living in rural areas were under represented compared with the general population. The distributions of the training, validation and test samples were similar, except for a slight under representation of rural populations in the test sample.

**Table 1:** Respondent characteristics.

Variable		Not imputed (*N* = 6148)			Imputed (*N* = 9324)*			Population		Chi-square test	
		Training	Validation	Test	Training	Validation	Test	Raw	Weighted	Vs. not imputed	Vs. imputed
***Categorical variables***	***Level***	n (%)	n (%)	n (%)	n (%)	n (%)	n (%)	%	%	p	p
Total	*-*	3699 (60.2)	1196 (19.5)	1253 (20.4)	5595 (60.0)	1871 (20.1)	1858 (19.9)	-	-		
Sex	*Female*	1940 (52.5)	616 (51.5)	637 (50.8)	2956 (52.8)	988 (52.8)	982 (52.9)	53.4	53.8	^a^ 0.107 ^b^ 0.117 ^c^ 0.038 ^d^ 0.514 ^e^ 0.254	^a^ 0.161 ^b^ 0.404 ^c0^.429 ^d^ 0.981 ^e^ 0.986
	*Male*	1759 (47.6)	580 (48.5)	616 (49.2)	2639 (47.2)	883 (47.2)	876 (47.2)	46.6	46.2		
Age group	*18–24 years*	244 (6.6)	74 (6.2)	85 (6.8)	401 (7.2)	134 (7.2)	132 (7.1)	10.6	8.8	^a^ <0.001 ^b^ <0.001 ^c^ <0.001 ^d^ 0.719 ^e^ 0.960	^a^ <0.001 ^b^ <0.001 ^c^ <0.001 ^d^ >0.999 ^e^ >0.999
	*25–34 years*	453 (12.3)	157 (13.1)	158 (12.6)	652 (11.7)	221 (11.8)	217 (11.7)	16.9	14.0		
	*35–44 years*	681 (18.4)	205 (17.1)	230 (18.4)	996 (17.8)	334 (17.9)	332 (17.9)	18.8	18.4		
	*45–54 years*	660 (17.8)	200 (16.7)	215 (17.2)	1002 (17.9)	333 (17.8)	334 (18.0)	15.5	17.0		
	*55–64 years*	713 (19.3)	249 (20.8)	248 (19.8)	1127 (20.1)	378 (20.2)	372 (20.0)	17.6	19.3		
	*65–74 years*	717 (19.4)	236 (19.7)	248 (19.8)	1080 (19.3)	362 (19.4)	360 (19.4)	11.6	12.8		
	*75–84 years*	210 (5.7)	68 (5.7)	62 (5.0)	298 (5.3)	98 (5.2)	99 (5.3)	6.9	7.6		
	*Over 85*	21 (0.6)	7 (0.6)	7 (0.6)	39 (0.7)	11 (0.6)	12 (0.7)	2.0	2.2		
Education	*Primary*	610 (16.5)	204 (17.1)	195 (15.6)	952 (17.0)	314 (16.8)	338 (18.2)	27.6	28.9	^a^ <0.001 ^b^ <0.001 ^c^ <0.001 ^d^ 0.489 ^e^ 0.675	^a^ <0.001 ^b^ <0.001 ^c^ <0.001 ^d^ 0.297 ^e^ 0.396
	*Secondary*	2270 (61.4)	714 (59.7)	777 (62.0)	3317 (59.3)	1085 (58.0)	1089 (58.6)	53.4	52.6		
	*Tertiary*	819 (22.1)	278 (23.4)	281 (22.4)	1326 (23.7)	472 (25.2)	431 (23.2)	19.0	18.4		
Residence	*Capital*	714 (19.3)	249 (20.8)	246 (19.6)	1116 (20.0)	389 (20.8)	385 (20.7)	18.1	17.9	^a^ 0.001 ^b^ 0.013 ^c^ <0.001 ^d^ 0.398 ^e^ 0.044	^a^ <0.001 ^b^ <0.001 ^c^ <0.001 ^d^ 0.653 ^e^ 0.030
	*Town*	1967 (53.2)	620 (51.8)	701 (56.0)	2978 (53.2)	983 (52.5)	1025 (55.2)	51.9	51.9		
	*Village*	1018 (27.5)	327 (27.3)	306 (24.4)	1501 (26.8)	499 (26.7)	448 (24.1)	30.0	30.1		
EQ-5D-5L index; *Mean (SD)*	*−0.848–1*	0.88 (0.20)	0.87 (0.22)	0.87 (0.22)	0.88 (0.20)	0.88 (0.21)	0.88 (0.21)	-	-	-	-

^a^ Training sample vs. weighted population, ^b^ Validation sample vs. weighted population, ^c^ Test sample vs. weighted population ^d^ Validation sample vs. train sample; ^e^ Test sample vs. train sample (Percentages may not add up to 100% due to rounding.) * Respondent characteristics were compared using data from one imputation.

#### Associations between variables

As observed in Scenario 2, the distribution of EQ-5D-5L index was distorted towards higher scores as the majority of values were between 0.75 and 1.00 (range: −0.848 to 1.000). When further analysing the distribution of the EQ-5D-5L index by continuous and categorical predictor variables, the highest scores (between 0.750 and 1.000) were observed for participants aged 40–70 years, married, living alone, living in a town or had secondary education. (Supplementary Fig. 1)

**Fig. 1:**
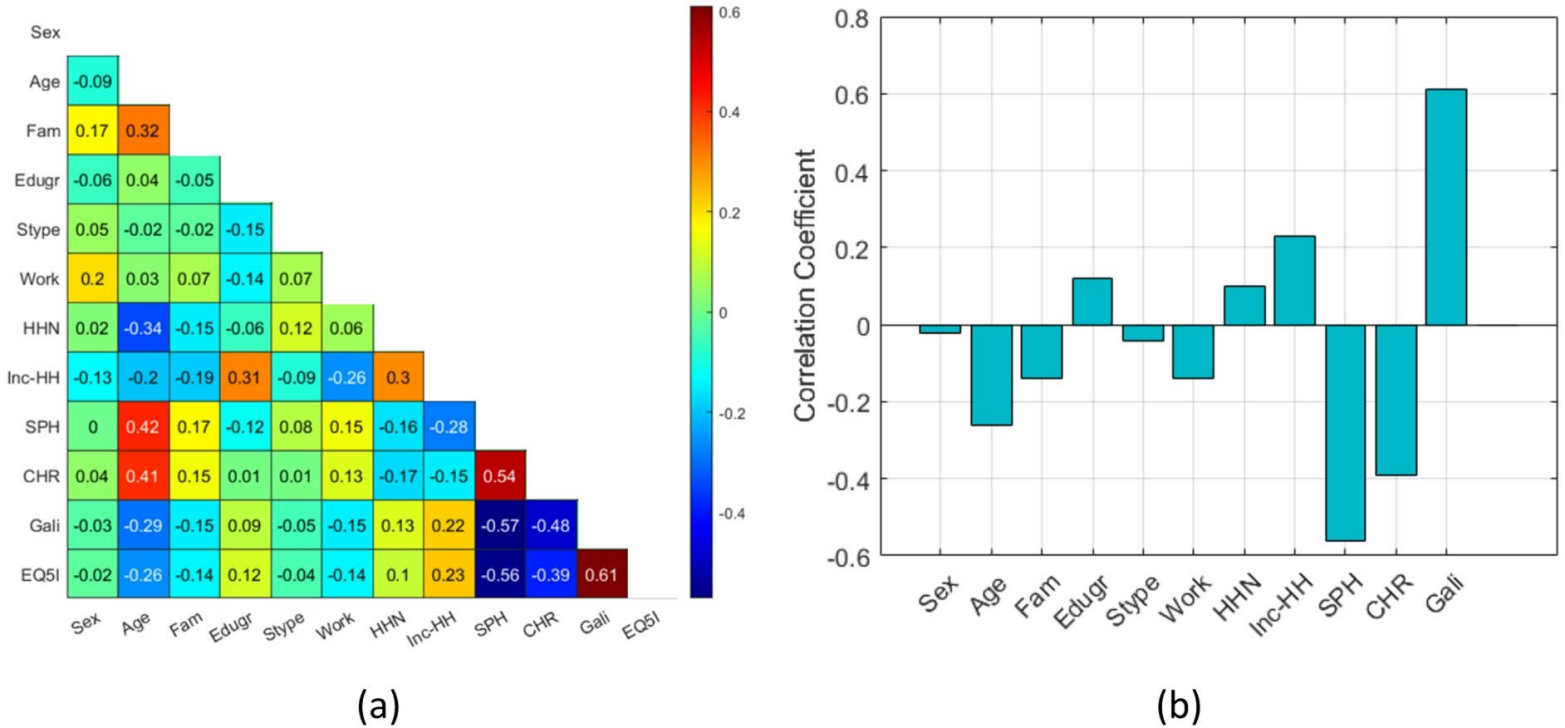
(**a**) Correlation matrix of variables (**b**) Correlation of predictor variables with the EQ-5D-5L index (Scenario 2, training dataset). Abbreviations: Fam – family status, Edugr – education level, Stype – settlement type, HHN – household number, Inc-HH – household income, SPH – self-perceived health, CHR – long-standing illness, Gali – Global Activity Limitation Indicator, EQ5I – EQ-5D-5L index.

The highest positive correlation was found between EQ-5D-5L index and the Global Activity Limitation Indicator (GALI; *r* = 0.61) item of the MEHM. Additionally, household income exhibited a moderate positive correlation with EQ-5D-5L index (*r* = 0.23). Conversely, self-perceived health (SPH) and the presence of a long standing illness (CHR) items of the MEHM measurement tool demonstrated the highest negative correlations (*r*=−0.56 and *r*=−0.39, respectively). Results are shown in Fig. 1.

#### Model selection

**Fig. 2:**
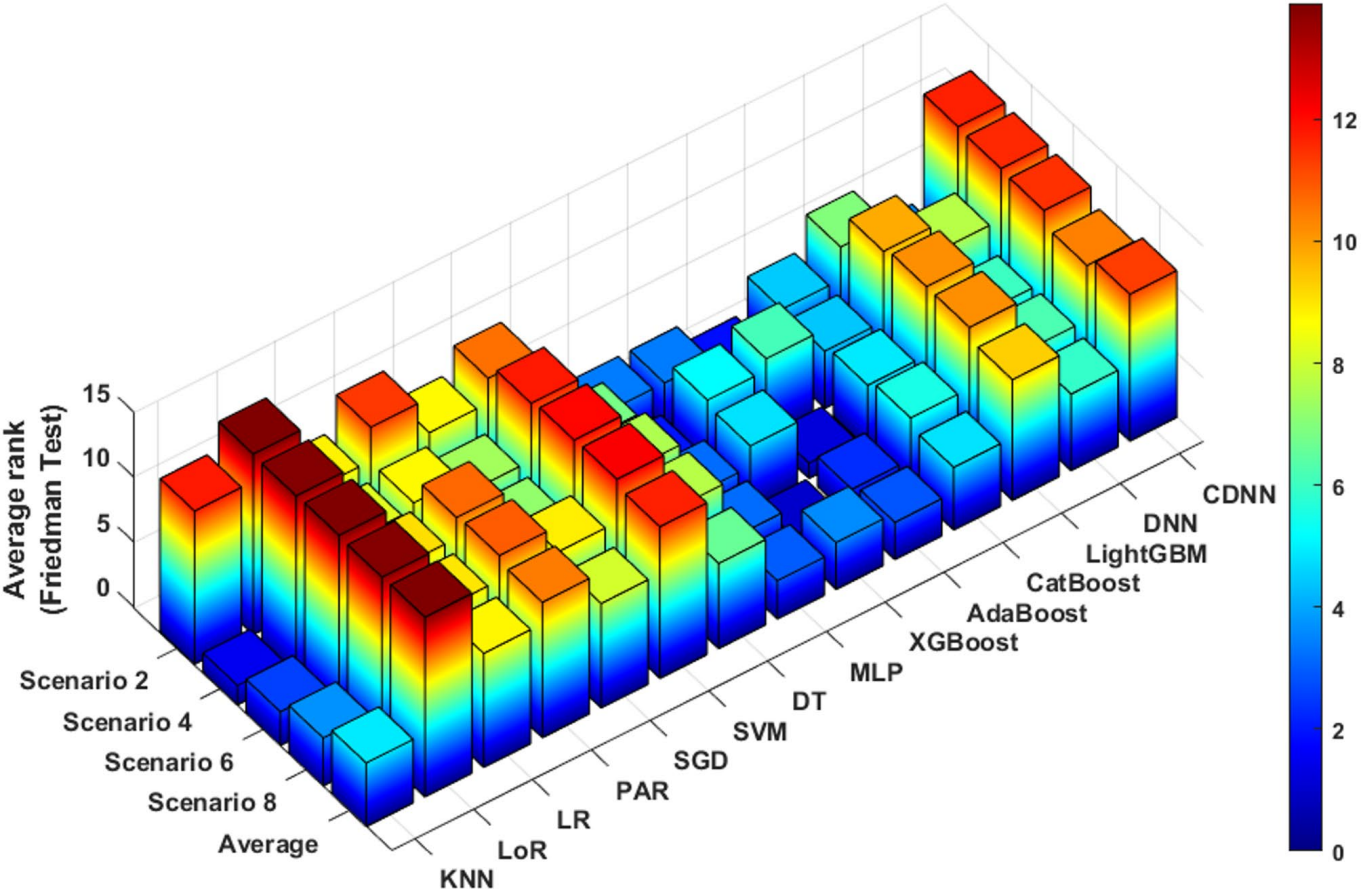
Results of the Friedman test for different ML models in Scenarios 2, 4, 6 and 8. Lower ranks indicate better performance compared with other models based on the G goodness metric (G score). Abbreviations: KNN - K-Nearest Neighbours, LoR - Logistic Regression, LR - Linear Regression, PAR - Passive Aggressive Regressor, SGD - Stochastic Gradient Descent, SVM - Support Vector Machine, DT - Decision Tree Regressor, MLP - Multilayer Perceptron, XGBoost - eXtreme Gradient Boosting regression, AdaBoost - Adaptive Boosting, CatBoost - Categorical Boosting, LightGBM - Light Gradient Boosting Machine, DNN - Deep Neural Network, CDNN - Convolutional Deep Neural Network.

The Friedman test revealed (χ²(13) = 45.98, *p* < 0.001) that out of all fourteen machine learning (ML) models analyzed, Adaptive Boosting (AdaBoost) achieved the highest average rank (2.87) across all Scenarios. It was followed closely by Multilayer Perceptron (MLP) and eXtreme Gradient Boosting regression (XGBoost), with average ranks of 2.95 and 3.6, respectively. Results are shown in Fig. 2.

### Performance of AdaBoost

**Fig. 3:**
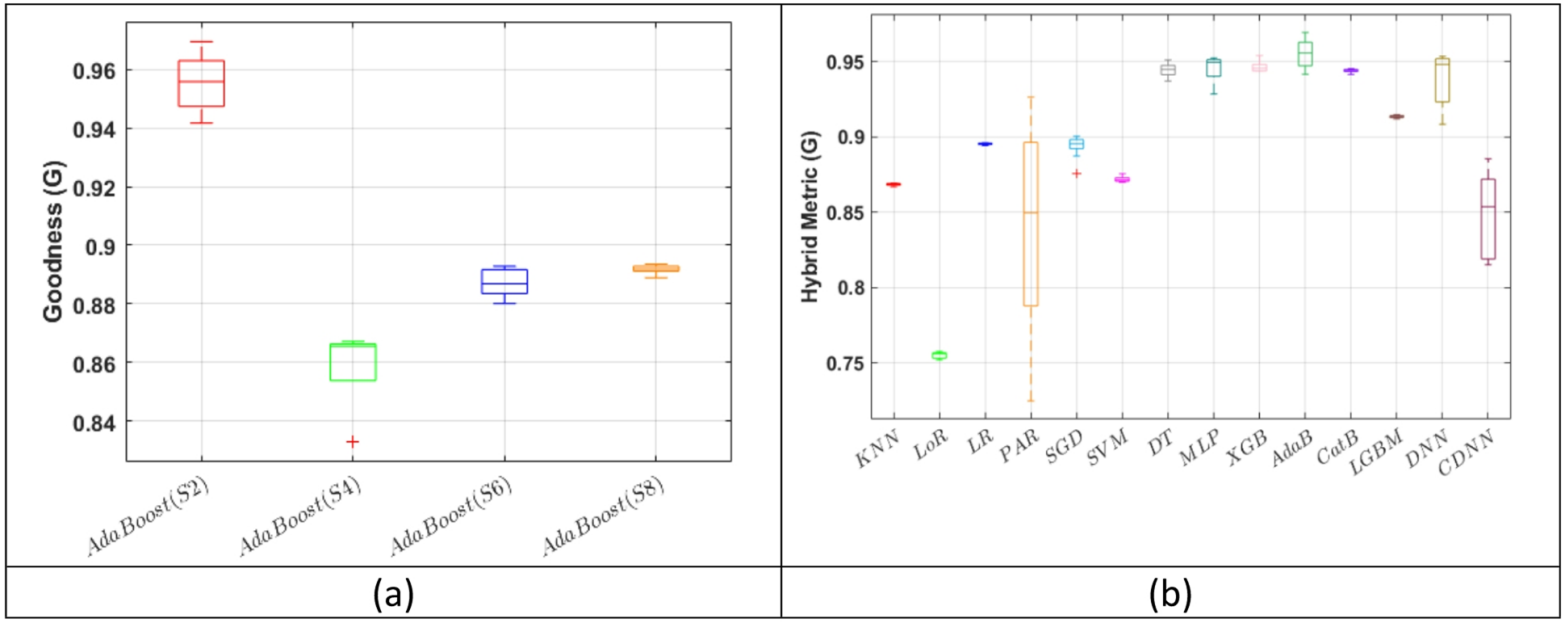
(**a**) The performance of AdaBoost across the four scenarios assessed with the G score, and (**b**) Comparison of different ML models in Scenario 2 in terms of G score. Results were obtained in the test datasets. Abbreviations: KNN - K-Nearest Neighbours, LoR - Logistic Regression, LR - Linear Regression, PAR - Passive Aggressive Regressor, SGD - Stochastic Gradient Descent, SVM - Support Vector Machine, DT - Decision Tree Regressor, MLP - Multilayer Perceptron, XGB - eXtreme Gradient Boosting regression, AdaB - Adaptive Boosting, CatB - Categorical Boosting, LGBM - Light Gradient Boosting Machine, DNN - Deep Neural Network, CDNN - Convolutional Deep Neural Network.

AdaBoost performed best in Scenario 2, where missing values were handled by deletion, achieving a score of 0.955 with the G goodness metric (G score; Fig. 3/a) and an R^2^ value of 0.465 (Supplementary Fig. 2). The G score is described in more detail in the Methods section by Eq. 1. Different weighting schemes applied during G-score calculation had no substantial effect, as AdaBoost consistently demonstrated the highest numerical performance across all models (Supplementary Table 2). In Scenario 2, XGBoost and MLP exhibited a similar yet slightly lower G score than AdaBoost (G = 0.947 and G = 0.946, respectively). (Fig. 3/b) Descriptive results for the performance metrics of all ML models in Scenarios 2, 4, 6 and 8 are presented in Supplementary Tables 3, 4, 5 and 6.

When comparing the performance of AdaBoost between the train and test dataset in Scenario 2, no overfitting was observed. Results are shown in Supplementary Fig. 3.

### Impact of the Minimum European Health Module (MEHM)

The effect of the MEHM on the prediction results was assessed by comparing the performance of AdaBoost between Scenario 2 (missing data deleted; demographic variables + MEHM) and Scenario 1 (missing data deleted; only demographic variables). It was observed that Scenario 2 outperformed Scenario 1. The highest, 35.5% increase was found for the fairness (F) metric (described in more detail in the Methods section), followed by a 8.4% improvement in the G score. A comprehensive overview of performance metrics for AdaBoost in Scenarios 1 and 2 is provided in Table 2. The graphical comparison of the results for the G score, MAE and R2 is shown in Supplementary Fig. 4.

**Table 2:** Comparison of the performance of AdaBoost between scenario 1 and 2.

AdaBoost (S2)								
Metric	RMSE	MAE	R2	F	RCA	RCB	MTAE	G
Min	0.160	0.097	0.450	0.863	0.961	0.998	0.067	0.942
Max	0.164	0.101	0.480	1.000	0.963	1.000	0.071	0.969
Mean	0.162	0.100	0.467	0.932	0.962	0.999	0.069	0.955
Median	0.161	0.100	0.470	0.933	0.962	0.999	0.070	0.956
STD	0.001	0.001	0.010	0.043	0.001	0.001	0.001	0.009
**AdaBoost (S1)**								
**Metric**	**RMSE**	**MAE**	**R2**	**F**	**RCA**	**RCB**	**MTAE**	**G**
Min	0.158	0.098	0.408	0.404	0.949	0.978	0.068	0.846
Max	0.173	0.123	0.450	0.684	0.963	0.997	0.092	0.882
Mean	0.165	0.109	0.430	0.577	0.957	0.987	0.079	0.871
Median	0.165	0.110	0.436	0.550	0.956	0.987	0.079	0.872
STD	0.007	0.011	0.019	0.098	0.006	0.009	0.011	0.012

Abbreviations: AdaBoost - Adaptive Boosting, RMSE – root mean square error, MAE – mean absolute error, F – fairness metric, RCA - relative clinical accuracy, RCB - relative clinical bias, MTAE – mean truncated absolute error, G – goodness metric, STD – standard deviation.

### Model optimization and feature selection

**Fig. 4:**
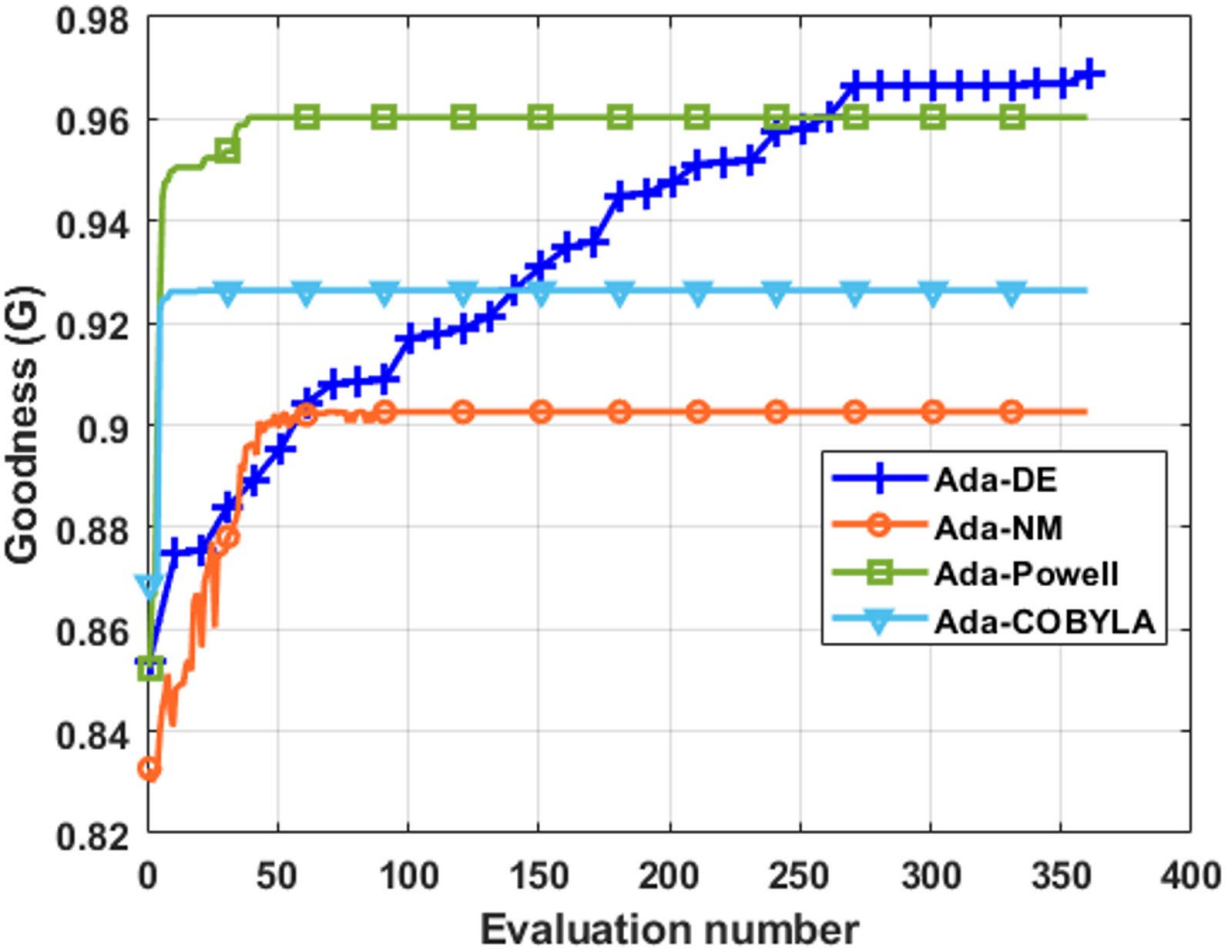
The convergence rate comparison for four hyper-parameters optimisation methods for AdaBoost in Scenario 2. Abbreviations: DE - Differential Evolution, NM - Nelder-Mead, COBYLA - Constrained Optimisation BY Linear Approximation.

Among the four optimization methods tested, Differential Evolution (DE) provided the best results, further improving the performance of AdaBoost by achieving a G score of 0.97. Results are shown in Fig. 4. The optimal settings were a learning rate of 0.79, three estimators, and a “square” loss function. Results of the Grid search approach are presented in Supplementary Fig. 5.

The performance of the Adaptive Elastic Net (AEN) feature selection technique was examined in ten independent runs. The convergence rate of AEN was remarkably fast and consistent across iterations, indicating its efficiency in improving model performance and optimizing the feature selection process (Supplementary Fig. 6/a and Fig. 6/b). Features in the best identified subset were age, work, household income and the three items of the MEHM (self-perceived health, presence of a long-standing illness, and the Global Activity Limitation Indicator). Furthermore, when AEN was incorporated in AdaBoost, a roughly 1.5% improvement in the G score was observed in Scenario 2 (Supplementary Fig. 6/c).

### Model interpretability and error analysis

**Fig. 5:**
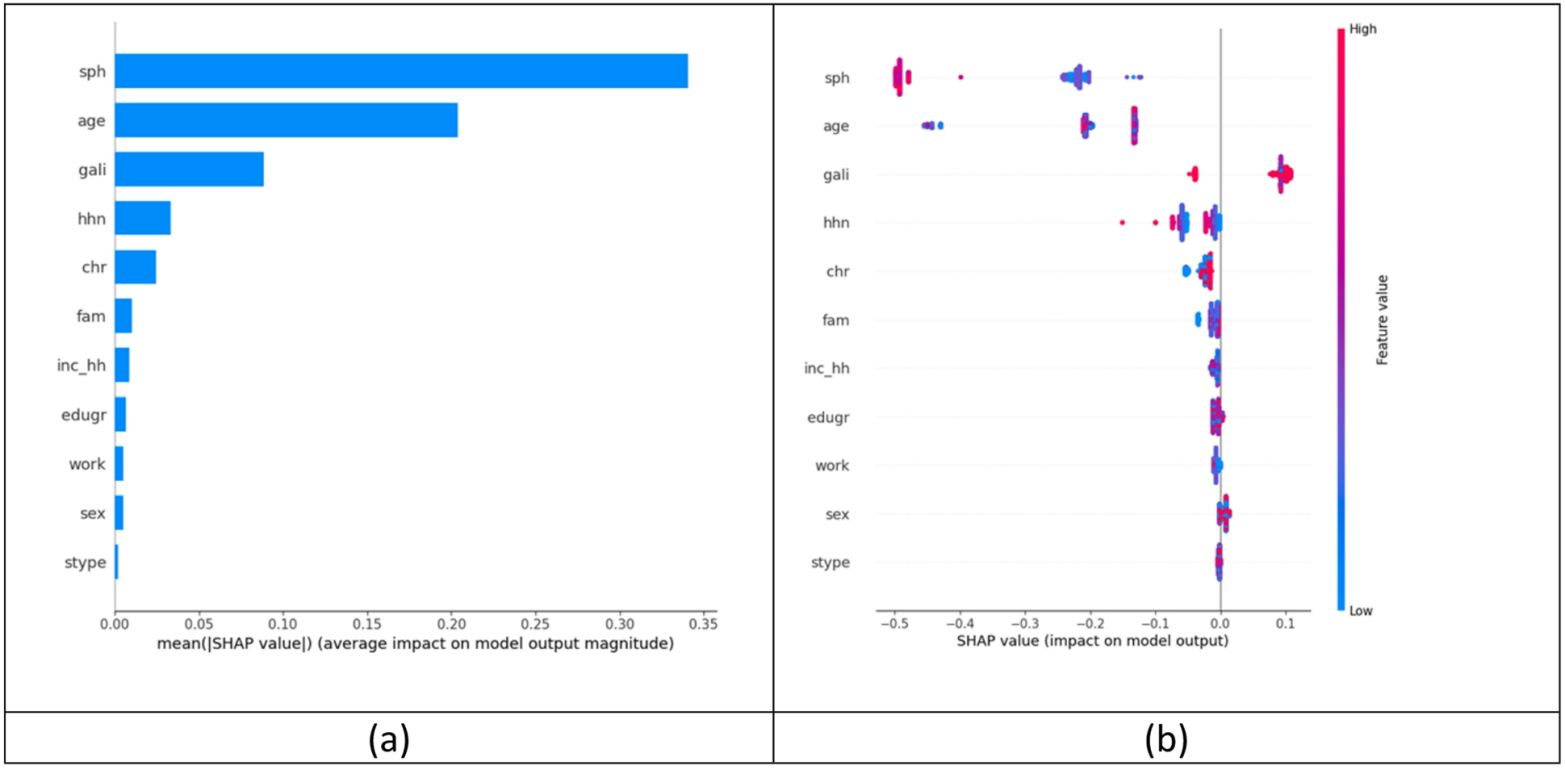
(**a**) mean absolute SHAP values, and (**b**) distribution of SHAP values by predictor variables. (Scenario 2, test dataset). Variables: SHAP - Shapley Additive Explanation, sph - self-perceived health, age – age, gali - Global Activity Limitation Indicator, hhn – household number, chr – long-standing illness, fam – family status, inc_hh – household income, edugr – education level, work – employment, sex – sex, stype – settlement type.

The variable with the most influence on the EQ-5D-5L estimation was ’self-perceived health’ (sph; 1st item of the MEHM), followed by age, and the Global Activity Limitation Indicator (gali; 3rd item of the MEHM). Results are shown on Fig. 5.

**Fig. 6:**
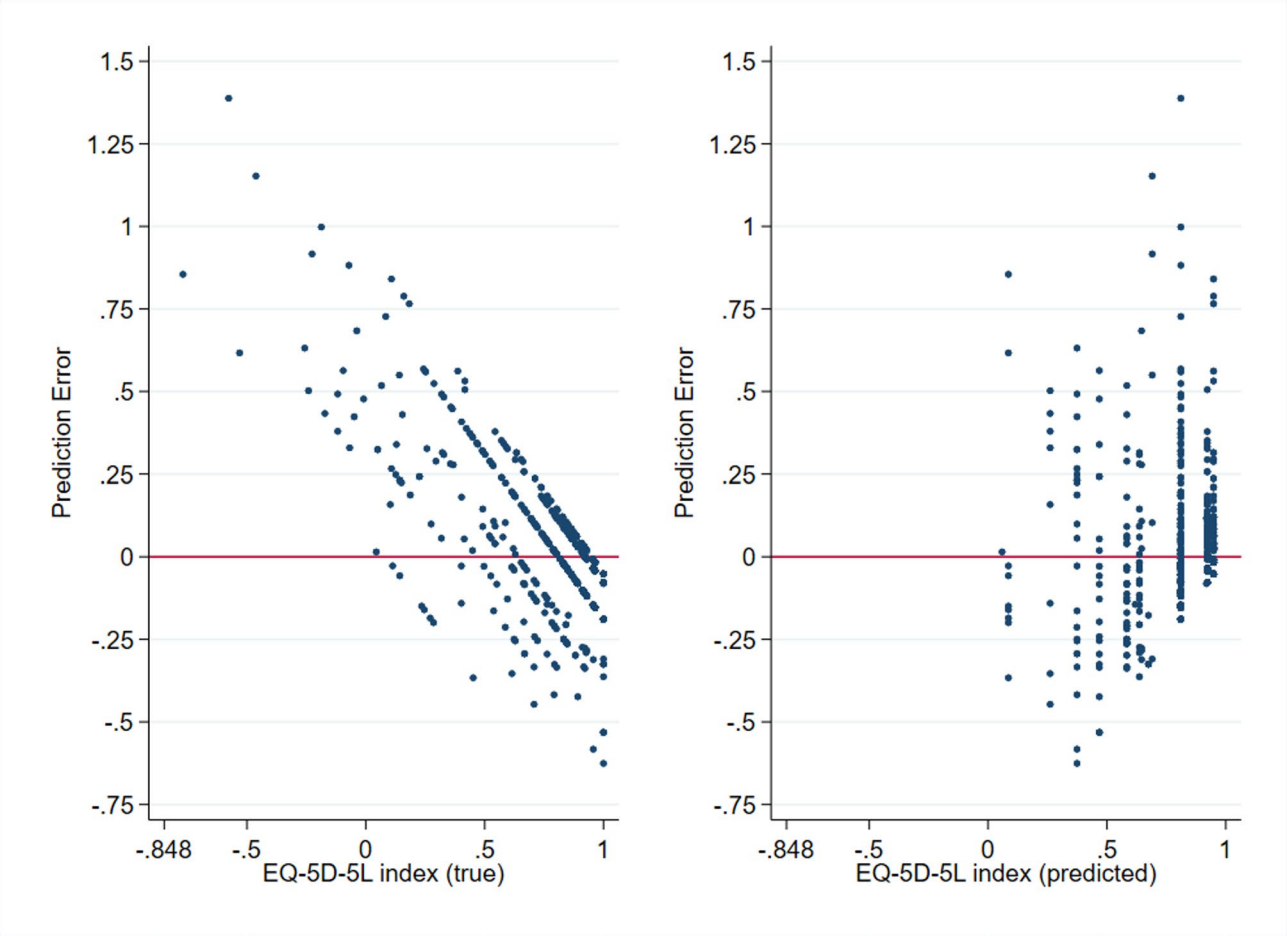
Distribution of prediction error by (**a**) true and (**b**) predicted EQ-5D-5L index values.

The error analysis revealed that the prediction error increased as the EQ-5D-5L index decreased and the error was positively biased in the lower score ranges. Results are shown in Fig. 6. Error was also significantly associated with sex, with women showing higher prediction errors than men (*p* = 0.025), but no significant associations were found with other key demographic variables such as age, education, or settlement type (Supplementary Fig. 7).

## Discussion

In this study, we successfully predicted EQ-5D-5L index values from a large, diverse general population dataset including basic sociodemographic characteristics and the Minimum European Health Module (MEHM). Our results showed that the inclusion of the MEHM in addition to sociodemographic variables translated into better performance in terms of fairness and bias of the estimation, although its effect on mean absolute error was negligible. We also found that missing data imputation had inferior effect on model performance compared to deletion. Among the fourteen machine learning (ML) models studied, Adaptive Boosting (AdaBoost) showed the best performance across all research scenarios, with high accuracy and G-score and potential for further improvement through optimized feature selection and hyperparameter tuning.

The use of ML to estimate utility values for preference-based patient-reported outcome measures (pPROM), such as the EQ-5D-5L, has shown an increasing trend in mapping studies^11^. To date, the majority of studies have relied on condition-specific non–preference-based PROMs (nPROMs) to predict utilities, focusing on areas such as Parkinson’s disease, functional disability and musculoskeletal disorders, and various cancers^17,18,25,26^. However, general nPROMs suitable for use in population samples have received less attention^15,27^. Our study sought to fill this gap by focusing on the MEHM, which has been relatively underutilized in ML-based mapping studies, despite being a widely used and standardized nPROM in statistical surveys across Europe. As demographic characteristics are routinely collected alongside the MEHM, we found it reasonable to incorporate basic demographic variables as predictors in the analysis, similarly to previous studies. Depending on the ML model, we observed an estimation accuracy ranging from 0.085 to 0.170 in terms of mean absolute error (MAE), which is comparable to what has been reported in previous nPROM studies using the PROMIS-GH10 and SF-12 measures^15,27^. These findings demonstrate that the MEHM can be used effectively for mapping EQ-5D-5L index values in population samples.

There is no clear consensus on which specific ML models are best suited for utility mapping. Previous studies have found that differences in performance between ML models are often marginal and largely dependent on the choice of evaluation metric, which typically focuses on mathematical accuracy, such as MAE or MSE^11,15,17^. In our study, model performance was primarily evaluated using the G metric, with AdaBoost achieved the best average rank across scenarios, closely followed by Multilayer Perceptron (MLP) and XGBoost. However, in terms of mean absolute error (MAE), a different order was observed, but the results in the best performing scenario (Scenario 2) were comparable to those previously reported in the literature for each model^14–16^. These findings underscore how challenging it is to select a single optimal model, given the small performance differences and the variability introduced by different evaluation metrics. We believe that in such cases, the actual numerical ranking of algorithms matters less than the overall reliability and the quality of the predictions that could be achieved with the available data. In our study, we placed particular emphasis on fairness. Specifically, we examined how much the predictions are biased against certain characteristics (often referred to as algorithmic bias), which is a topic receiving increasing attention in ML research^28^. Therefore, we adopted the G metric as our primary performance measure^29^. Although this choice offers a novel approach to performance evaluation, it currently limits cross-study comparability as experience with the G metric is still limited.

The issue of fairness and algorithmic bias is also an area of research in mapping studies. It has been reported that the size of prediction error is not evenly distributed in the EQ-5D-3L and the EQ-5D-5L index ranges as it tends to increase towards lower values^14,30–32^. Therefore, we also considered it relevant to compare ML models from this perspective and to evaluate the role and impact of the MEHM on the fairness of predictions. Our results showed that the inclusion of MEHM significantly improved the imbalance and the fairness of the prediction as out of all performance metrics studied, the highest improvement was observed for the F metric. Also, the MEHM moderately increased the G score, although no meaningful improvement was found in case of other classical performance metrics such as MAE, R2 and root mean squared error (RMSE). These results indicate that the information content of the MEHM significantly contributed to the more even dispersion of estimation error across the whole EQ-5D-5L index range. However, the error analysis showed that the prediction error increased towards lower EQ-5D-5L index values. Also, the error was systematically biased in the positive direction meaning higher predicted EQ-5D-5L index for those in poorer health condition. In terms of feature importance, the study also provides important quantitative information about the contribution of the MEHM items as self-perceived health (SPH) and the global activity limitation indicator (GALI) were identified as the first and third most influential predictor variables in estimating the EQ-5D-5L index values.

Missing data represent a common problem in pPROM estimation studies^33^. For example, Zrubka and colleagues used the indicator method to handle missing data when predicting EQ-5D-3L index values. ^14^Our study adds to the body of literature as we tested different missing data handling strategies, including multiple imputation by chained equations (MICE), which is a state-of-the-art technique in ML studies^24,34^. Our results confirmed that the MICE is effective when predicting EQ-5D-5L values using ML methods. However, we observed the best performance of AdaBoost when missing data was handled with deletion (Scenario 2). A possible explanation to this phenomenon is that MICE relies heavily on the associations between variables, using observed data to predict missing values. In our study, although missing data patterns and mechanisms justified the use of MICE, the relatively small number of variables limited the available information about interactions and may have constrained the accuracy of the imputations. We believe this explains why using less but more robust data achieved more accurate and less biased predictions than a larger dataset containing greater uncertainty due to imputation (despite its obvious efficiency). We believe this is an important finding, as it highlights key caveats of MICE and provides useful guidance for researchers considering options for handling missing data in ML studies. Although MICE was found to be inferior in the present study compared to missing data deletion, more advanced procedures such as ML–based imputation methods are now available that warrant investigation in the future to test whether they offer a better performance in the context of EQ-5D-5L mapping from statistical health data.

A key limitation of our study is the absence of external validation. However, we trained the models on a large, nationally representative sample and reserved a holdout dataset for testing. While model performance may vary in future studies or in datasets with different sampling frames or morbidity profiles, we believe our approach supports generalizability within similar settings. Moreover, given the broad demographic similarities across Central and Eastern Europe and the availability of our predictor set (MEHM items and basic sociodemographics) in many European countries, we expect reasonable transportability within the region. Nonetheless, formal local testing remains essential, especially for non-population samples or for populations that differ markedly in cultural or socioeconomic characteristics. A second limitation is that participants were recruited using a non-probability sampling procedure (quota sampling), a widely used and accepted method in population surveys. Given that the distributions of key demographic characteristics in our sample (sex, age, education, and place of residence) closely matched those observed in the general population (considered as the target population for the analysis) the sampling method is not expected to compromise the validity of the results, and our conclusions should be generally applicable to new population samples. Moreover, the wide range of EQ-5D-5L index scores observed indicates that individuals across a broad spectrum of health are represented in our study. Nevertheless, as quota sampling may introduce unmeasured selection biases, caution is warranted when extending conclusions to demographically different populations. Another limitation is that the G metric used in this study to evaluate model performance is a relatively new indicator, and currently there is limited empirical experience with its application. However, the results of the sensitivity analysis showed that alternative weights did not meaningfully change the relative ranking of the ML models and alter our main findings. Therefore, we consider the G metric an important and promising step toward developing a composite measure that supports a more multidimensional assessment of ML model performance. A further limitation is that despite the apparent improvement when including the MEHM as a predictor, there was still a high imbalance of estimation error observed. A possible explanation is that individuals with perfect or nearly perfect health were present at a higher proportion - which is typical of population samples - so the ML algorithm, despite having a very good average error, could not predict poor health with sufficient accuracy. Future studies should therefore focus on reducing the imbalance and increasing the fairness of the estimation, since in a real-life scenario the goal would be to predict the health status of the ill individuals within an acceptable error range. A possible way to overcome this problem, and also a great avenue for future research, is to examine how these sample inequalities could be treated, for example with different weighting methods or involving samples of specific patient groups.

Our study has important health-policy and decision-making implications. Health-technology-assessment (HTA) agencies, such as the National Institute for Health and Care Excellence (NICE), accept mapped utilities when direct EQ-5D data are unavailable or cannot be collected, provided that the resulting uncertainty is examined and reported through sensitivity analyses^35^. ML-based mapping can therefore serve as a useful adjunct, as it leverages routinely collected data to populate cost-effectiveness models rapidly. However, the algorithmic bias found in our study and also in previous works (most notably the over-prediction of utilities in poor health states) can attenuate incremental QALY gains and inflate ICERs for high-burden populations, thereby threatening the validity of cost-effectiveness analyses and leading to less favourable decisions^36^. Nonetheless, in real-world scenarios, it remains feasible to identify the superior alternative between different options (except in cases of marginal health gains), and dominant cost-effective cases generally do not pose a problem either. However, precise interpretation of results in relation to the willingness-to-pay threshold remains a challenge and represents one of the main limitations of using estimated utility values. Therefore, decision-makers must be aware of these risks and actively mitigate them, for example by subgroup-specific calibration of utilities or by forcing modellers to conduct appropriate sensitivity analyses. All in all, we believe that direct EQ-5D data collection should remain the gold standard whenever it is feasible, whereas ML-based utility estimates should be viewed as a pragmatic fallback when questionnaire length, cost, or a retrospective study design precludes primary data collection.

In conclusion, we successfully predicted EQ-5D-5L index values using cutting-edge ML models from basic sociodemographic and health data in a large and diverse dataset of extensive population surveys. Our results showed that the Minimum European Health Module (MEHM) can improve model performance by increasing the fairness and reducing the imbalance of prediction error across the whole EQ-5D-5L index range. However, the significant residual bias indicates that the issue of prediction fairness, which is of high clinical importance, is still present. Therefore, further studies are warranted to test possible methods for its reduction, such as different weighting schemes or severity-stratified calibration. Compared to imputation, better results were observed when missing data was handled with deletion, highlighting the importance of data quality when predicting HRQoL data with ML-based methods. Nevertheless, direct EQ-5D-5L measurement should remain the gold standard wherever feasible, but our results demonstrate that ML mapping offers a viable fallback when primary data collection is impractical or impossible. Our results can aid researchers, analysts, and policy-makers seeking rapid and transparent ways to populate early cost-effectiveness models, scenario analyses, or retrospective studies when direct utility data are unavailable.

## Methods

### Data collection

Between 2019 and 2021, we conducted seven cross-sectional surveys among the Hungarian adult general population. Five studies included respondents aged ≥18 years, while two studies included respondents aged ≥40 years (Supplementary Table 7)^37–52^. To ensure representativeness, study samples were selected by quota sampling, a non-probability method. Quotas for sex, age, education, and settlement type were set in advance so that participants were recruited in proportions matching the general population along these key variables. All respondents provided written informed consent upon enrolment and participation was voluntary, and anonymous. For the purpose of the present research, data from the seven studies were merged into a joint database. We obtained ethical approval from the Medical Research Council of Hungary (IV/8070-1/2020/EKU; BM/7790-1/2023) for the secondary analysis of the merged dataset. The studies included were also approved by the Medical Research Council of Hungary, details are provided in the original manuscripts. No further consent was needed from participants to be included in the secondary analysis. All methods were carried out in accordance with the relevant guidelines and regulations and with the principles of the Declaration of Helsinki.

### Main variables

EQ-5D-5L ^9^ and MEHM^13^ were administered to all participants, along with basic sociodemographic characteristics (sex, age, education, settlement type, marital status, employment, number of household members, and income). EQ-5D-5L index values (utilities) were calculated using the Hungarian value set, with values ranging from 1 (perfect health) to − 0.848 (extreme problems in all five dimensions)^53^. The minimum clinically important difference (MCID) in the Hungarian EQ-5D-5L utility values is 0.066 ^29^. The recorded variables by study and the proportion of missing data are shown in Supplementary Table 1. EQ-5D-5L and MEHM are introduced in Supplementary Table 8.

### Prediction scenarios and study workflow

Eight prediction scenarios were developed to determine the contribution of the MEHM to prediction accuracy and to assess the effect of data imputation. In scenarios 1, 3, 5 and 7, only the demographic variables were included as predictors and four different missing data handling strategies were used (one in each scenario; introduced in the next section), while scenarios 2, 4, 6 and 8 included both the MEHM and the demographics as predictors and used the same four missing data handling strategies. An overview of the eight scenarios is provided in Supplementary Table 9.

Out of these eight scenarios, five were analysed with machine learning. First, to test the effect of imputation and select the data handling strategy achieving the best results, model training and initial performance evaluation were carried out in scenarios 2, 4, 6 and 8, where all predictors were included. In the next phase, we analyzed the scenario that used the best data-handling strategy and included only the demographics as predictors (either scenario 1, 3, 5 or 7) and compared the results to its counterpart in order to determine the contribution of the MEHM to model performance and prediction accuracy.

### Data management

#### Inclusion criteria

Respondents with available EQ-5D-5L utility values were included in the joint database. Variables from the seven surveys were recoded using uniform rules (Supplementary Table 1). Data management was performed in Stata 17 (StataCorp LCC., College Station, TX, USA).

### Data splitting

The joint database was stratified by sex and age and randomly split into training, validation and test datasets in a 60%, 20% and 20% ratio, respectively. The test datasets were not shared with the analyst team until the completion of model training to prevent information leakage. Furthermore, in Scenarios 4, 6 and 8, training and validation datasets were separated before the imputation of missing data.

### Handling missing data

Missing data exhibited a non-monotonic, multivariate pattern. Reasons for missingness included omission from the entire survey, skip logic due to irrelevant response options, or the option to decline response for sensitive items (e.g., income, subjective health). We treated non-recorded variables as missing completely at random, and nonresponses concerning income as missing at random. We compared four missing data handling strategies (Supplementary Table 9). First, we deleted observations with missing values from the datasets (applied in Scenarios 1 and 2). Second, imputations were performed without including EQ-5D-5L in of any of the datasets (Scenarios 3 and 4); third, EQ-5D-5L index was used in the imputation of the training dataset but not the validation and test datasets (Scenarios 5 and 6); and fourth, the EQ-5D-5L index was used in the imputation of the training and the validation datasets but not the test dataset (Scenarios 7 and 8). Imputations were performed via the multiple imputation by the chained equations (MICE) method performing 30 imputations^54^. MICE iteratively fits a series of regression models, each modeling one variable’s missing values conditional on all other variables, under the assumption that each model correctly specifies the relationship between the target variable (with missing entries) and its predictors. The imputation was attempted with predictive mean matching (PMM), using the 10 nearest neighbour observations to the linear prediction of the missing value. The training, validation and test datasets were imputed separately.

### Data transformations

Data were normalized using the Standard Scaler technique, a standard approach for normalization in the field of machine learning^55^.

### Descriptive statistics

We compared the distribution of key sociodemographic variables (sex, age group, education, type of residence) of the training, validation and test datasets against that of the general population using the 2011 Census data, which was available at the time of data collection^56^. We tabulated the raw and weighted population data to reflect the age composition of the samples. Using chi-square tests, we compared the train, validation, and test samples with the weighted population and within both imputed and non-imputed datasets, we compared the validation and test samples to the train sample. In scenario 2, we assessed and graphed the distribution of all variables in the training dataset and examined the distribution of the EQ-5D-5L index by continuous and categorical predictors. We also calculated and graphed the correlation matrix of all predictors.

### ML methods

#### Included models

In a previous study using RWD for the prediction of utilites values for the 3-level version of the EQ-5D, eXtreme Gradient Boosting regression (XGBoost) provided the best predictive performance^14^. Therefore, we considered this method as the baseline, and included the following algorithms in this study: K-Nearest Neighbours (KNN), Logistic Regression (LoR), Linear Regression (LR), Decision Tree Regressor (DT), Multilayer Perceptron (MLP), Passive Aggressive Regressor (PAR), Support Vector Machine (SVM), Stochastic Gradient Descent (SGD), Adaptive Boosting (AdaBoost), Categorical Boosting (CatBoost), Light Gradient Boosting Machine (LightGBM), Deep Neural Network (DNN), and Convolutional Deep Neural Network (CDNN). Technical details of the models are provided in Supplementary Table 10.

#### Applied performance metrics

Although we monitored model performance via a broad range of performance metrics, a single goodness metric, G was used for model optimisation and performance evaluation. In addition to G and its components, we computed mean square error (MSE), root mean squared error (RMSE), mean absolute error (MAE) and the Pearson correlation coefficient (r). To reflect the magnitude of clinically relevant errors, we computed mean truncated absolute error (MTAE). Formulas are provided in Supplementary Table 11. Clinical relevance was determined on the basis of MAE.

#### The G goodness metric

G combines clinically relevant accuracy, bias, and fairness in a single metric on the 0–1 interval, where values closer to 1 represent better model performance^29^. G assumes that within the true value ±1/2 MCID range (i.e., clinically irrelevant error), differences between patients are not perceivable, and predictions can be considered clinically as accurate, unbiased and fair. Hence, G is computed from truncated errors falling outside the clinically irrelevant error range. All components of G, namely relative clinical accuracy (RCA), relative clinical bias (RCB), and fairness (F) are projected on the 0–1 interval. RCA and RCB reflect truncated errors relative to the maximum perceivable errors in the range of EQ-5D-5L. F reflects the association of truncated prediction errors with a selected set of background variables (called protected variables). This relationship is quantified using the Predictive Power Score (PPS; range 0–1), which can detect both linear and non-linear relationships^57^. F -is calculated by taking the complement (1 minus PPS) for each protected variable and then averaging these values. Thus, an F score of 0 indicates a completely unfair prediction (errors fully depend on protected variables), while an F score of 1 indicates perfect fairness (no association between errors and protected variables). Components of G and details of their calculation are shown in Supplementary Table 11. The formula of G is shown in Eq. 1, where w denotes the importance weight of the fairness component.

1 $$\:G\:=\:wRCA \times RCB\:+\:(1-w) \times F,\:with\:G,\:RCA,\:RCB,\:F,\:w\:\in\:\:\left[\mathrm{0,1}\right]$$

Technical details of G are provided in the original publication^29^. In this study we used an importance weight of 0.8 to calculate G, and EQ-5D-5L utilities, age, sex, education, income, and working status as protected variables to calculate F.

#### Statistical tests for model selection and performance evaluation

The best model was selected using the Friedman test based on the rank of G scores over scenarios 2, 4, 6, and 8^58^. We also performed a sensitivity analysis to assess how alternative weighting schemes influence the computed G scores and the models’ relative performance. To detect overfit, the performance of models in the training and test datasets were compared in terms of G, MAE, R^2^ and the RMSE.

We quantified the contribution of the MEHM to prediction accuracy by comparing the G scores, the mean absolute error (MAE) and R^2^ values of the best performing ML model between scenarios 1 and 2.

#### Model optimisation

We aimed to fine-tune the best performing ML model, AdaBoost, using various optimization techniques. The optimal hyper-parameter configuration was identified using Grid search^59^. Optimization was carried out using four methods: Differential Evolution (DE), Nelder-Mead (NM), Powell method, and COBYLA method (Constrained Optimisation BY Linear Approximation)^60–63^.

#### Feature selection

To select the most relevant features for the best performing model, a hybrid feature selection technique was used, combining Elastic-net^64^ with a fast local search known as 1 + 1EA which is an efficient strategy to adjust the parameters of Elastic Net, including L1-ratio (determining the balance between L1 and L2 regularization), alpha (controlling the strength of regularization), and different selection methods. This technique uses a heuristic approach, incrementally refining the configuration of Elastic Net iteratively until a local optimum is reached.

### Model examination and error analysis

We examined prediction results of the optimal model on the test dataset using Shapley Additive Explanation (SHAP)^65^. For each observation, SHAP values measure the (positive or negative) contribution of each predictor variable in explaining the variation between a predicted EQ-5D-5L utility and the average value of the utility across the entire dataset. SHAP is a model-agnostic post-hoc interpretation framework, which allows model examination at the level of both individual observations (local interpretability) and the entire model (global interpretability).

For global model interpretation, we plotted feature importance plots and beeswarm plots for the included variables in each prediction scenario. Feature importance plots are simple bar charts of the mean absolute SHAP values in the dataset. For each predictor, beeswarm plots depict the SHAP values of each observation reflecting their density, providing detailed insight about the relationship between the predictor and outcome.

Error analysis was performed by calculating the absolute error of each prediction obtained with the best performing, optimized model and assessing the error distribution by the main demographic variables (sex, age, education, type of settlement), and the true and predicted EQ-5D-5L index values.

### Model reproducibility

In the study, Python 3.9.16 was utilized as the programming language. Several open-source Python libraries were employed, including Scikit-learn (0.23.2), NumPy (1.19.1), Pandas (1.1.0), SciPy (1.5.2), and TensorFlow (2.3.0). Scikit-learn played a central role in providing a wide range of machine learning models used in this study. Additionally, ensemble models such as XGBoost, LightGBM, and CatBoost were employed, which are open-source platforms that are publicly available. TensorFlow, an open-source machine learning framework developed by Google, was used specifically for implementing deep neural networks (DNN) and convolutional deep neural networks (CDNN). The AdaBoost algorithm was implemented using the Python Scikit-learn ensemble library. An AdaBoost regressor, a meta-estimator, was employed in the process. Initially, a regressor was fitted on the original dataset, followed by multiple iterations where additional regressors were fitted on the same dataset, adjusting the weights of instances based on the error of the current prediction. This adaptive approach allowed subsequent regressors to focus more on challenging cases, enhancing the overall predictive performance. For data encoding, two scaling techniques, StandardScaler and MinMaxScaler, were tested to normalize the input features effectively. To validate the model, a robust 10-fold cross-validation method was utilized, ensuring reliable performance assessment across different subsets of the data. Furthermore, hyperparameter tuning was conducted using the Differential Evolution (DE) optimization method, optimizing the model’s parameters for enhanced predictive accuracy and generalizability.

## Supplementary Information

Below is the link to the electronic supplementary material.

Supplementary Material 1

## Data Availability

A randomly selected 10% of the dataset analysed in the current study and the code that was used to develop and assess the adaptive boosting ensemble model are available at Zenodo repository: https://zenodo.org/uploads/13953060?token=eyJhbGciOiJIUzUxMiJ9.eyJpZCI6ImFiMGEyMjI3LTViNTEtNGM1OS1hNzc5LWQyZGQ5NmE0ZDUyOSIsImRhdGEiOnt9LCJyYW5kb20iOiJmNzE2NTY4OTJiYzIyZDQzNWMxZjllNmRlYTIxZWRjNCJ9.kP_tcBY5W4TTOt5Zq5UWf5uZc6f0tbEfxTgJj9pUJ2Aw1_LxZaBGnXjdGXaGofQMQHWRA9D42C2C2-PIuGRXGg.
