# Supplementary material for: Improving the value of population health data for health policy and decision-making using machine learning algorithms in EQ-5D-5L index estimation
Source: Sci Rep. 2026 Jan 30;16:4329. doi: 10.1038/s41598-025-32123-6 (PMC12865033; doi:10.1038/s41598-025-32123-6)
Supplement: Supplementary file 1 — Supplementary Material 1 [file 41598_2025_32123_MOESM1_ESM.docx]

**SUPPLEMENTARY MATERIALS**

**Tables**

**Supplementary Table 1**. Number of complete observations by variables and studies

|  | Variable | | | | Study | | | | | | | Missing (%) |
| --- | --- | --- | --- | --- | --- | --- | --- | --- | --- | --- | --- | --- |
|  | # | Name | Description | Code | 1 | 2 | 3 | 4 | 5 | 6 | 7 |  |
| Demographic variables | 1 | EQ-5D-5L utility | continuous; range [-0.848, 1] | eq5i | X | X | X | X | X | X | X | 0% |
|  | 2 | Sex | male / female | sex | X | X | X | X | X | X | X | 0% |
|  | 3 | Age | continuous; years | age | X | X | X | X | X | X | X | 0% |
|  | 4 | Marital status | single / married / domestic partnership / divorced / widowed / other | fam | X | X | X | X | X | X | X | 0% |
|  | 5 | Education | primary / secondary / tertiary | edugr | X | X | X | X | X | X | X | 0% |
|  | 6 | Settlement type | capital / town / village | stype | X | X | X | X | X | X | X | 0% |
|  | 7 | Employment | full time / part time / pensioner / disability pensioner / student / unemployed (seeking) / unemployed (not seeking) / housemaker / other | work | 0 | X | X | X | X | X | X | 10% |
|  | 8 | Number of household members | continuous; the number of people living in respondent’s household | hhn | X | X | X | X | X | X | X | 0% |
|  | 9 | Household income | continuous; EUR | inc_hh | X | X | X | X | X | X | X | 22% |
| MEHM | 10 | Self-perceived health | very good / good / fair / bad / very bad | sph | X | X | X | X | X | X | X | 0.1% |
|  | 11 | Long-standing illness | yes / no | chr | X | X | X | X | X | X | X | 5% |
|  | 12 | Global Activity Limitation Indicator (GALI) | not limited / limited but not severely / severely limited | gali | X | X | X | X | X | X | X | 2% |

The 3 variables related to MEHM are denoted with sph, chr and gali.

The percentage of missing values for a given variable is denoted in the ’Missing (%)’ column.

**Supplementary Table 2.** Impact of weighting in G-score calculation on model performance (Scenario 2)

| **Model** | **Metrics from Scenario 2** | | | **G=(RCA*RCB*w)+((1-w)*F)** | | | | | | | | | | | | |
| --- | --- | --- | --- | --- | --- | --- | --- | --- | --- | --- | --- | --- | --- | --- | --- | --- |
|  | **RCA** | **RCB** | **F** | **w = 0** | | **w = 0.2** | | **w = 0.4** | | **w = 0.6** | | **w = 0.8** | | **w = 1** | |  |
|  |  |  |  | **G** | **Rank** | **G** | **Rank** | **G** | **Rank** | **G** | **Rank** | **G** | **Rank** | **G** | **Rank** |  |
| CatBoost | 0.967 | 0.991 | 0.888 | 0.888 | 5 | 0.902 | 5 | 0.916 | 5 | 0.930 | 5 | 0.944 | 5 | 0.958 | 2 |  |
| AdaBoost | 0.962 | 0.999 | 0.932 | 0.932 | 1 | 0.938 | 1 | 0.944 | 1 | 0.949 | 1 | 0.955 | 1 | 0.961 | 1 |  |
| XGBoost | 0.965 | 0.993 | 0.905 | 0.905 | 3 | 0.916 | 3 | 0.926 | 3 | 0.937 | 3 | 0.948 | 2 | 0.958 | 3 |  |
| SVM | 0.957 | 0.997 | 0.543 | 0.543 | 11 | 0.625 | 11 | 0.707 | 11 | 0.790 | 10 | 0.872 | 10 | 0.954 | 9 |  |
| DT | 0.95 | 0.999 | 0.928 | 0.928 | 2 | 0.932 | 2 | 0.936 | 2 | 0.941 | 2 | 0.945 | 4 | 0.949 | 11 |  |
| LightGBM | 0.966 | 0.991 | 0.74 | 0.740 | 7 | 0.783 | 7 | 0.827 | 7 | 0.870 | 7 | 0.914 | 7 | 0.957 | 4 |  |
| KNN | 0.965 | 0.985 | 0.539 | 0.539 | 12 | 0.621 | 12 | 0.704 | 12 | 0.786 | 11 | 0.868 | 11 | 0.951 | 10 |  |
| SGD | 0.962 | 0.993 | 0.649 | 0.649 | 9 | 0.710 | 9 | 0.772 | 9 | 0.833 | 9 | 0.894 | 9 | 0.955 | 7 |  |
| LoR | 0.957 | 0.959 | 0.109 | 0.109 | 14 | 0.271 | 14 | 0.433 | 14 | 0.594 | 14 | 0.756 | 14 | 0.918 | 13 |  |
| LR | 0.962 | 0.993 | 0.657 | 0.657 | 8 | 0.717 | 8 | 0.776 | 8 | 0.836 | 8 | 0.896 | 8 | 0.955 | 7 |  |
| MLP | 0.966 | 0.991 | 0.898 | 0.898 | 4 | 0.910 | 4 | 0.922 | 4 | 0.934 | 4 | 0.945 | 3 | 0.957 | 4 |  |
| PAR | 0.934 | 0.959 | 0.606 | 0.606 | 10 | 0.664 | 10 | 0.722 | 10 | 0.780 | 12 | 0.838 | 13 | 0.896 | 14 |  |
| DNN | 0.965 | 0.992 | 0.865 | 0.865 | 6 | 0.883 | 6 | 0.902 | 6 | 0.920 | 6 | 0.939 | 6 | 0.957 | 6 |  |
| CDNN | 0.949 | 0.988 | 0.493 | 0.493 | 13 | 0.582 | 13 | 0.671 | 13 | 0.760 | 13 | 0.849 | 12 | 0.938 | 12 |  |

Abbreviations: RCA – Relative Clinical Accuracy, RCB – Relative Clinical Bias, F – Fairness, w – weight, CatBoost – Categorical Boosting, AdaBoost – Adaptive Boosting, XGBoost – eXtreme Gradient Boosting, SVM – Support Vecor Machine, DT – Decision Tree Regressor, LightGBM – Light Gradient Boosting, KNN – Nearest Neighbors, SGD – Stochastic gradient descent, LoR – Logistic Regression, LR – Linear Regression, MLP – Multilayer Perceptron, PAR – Passive Aggressive Regressor,DNN – Deep Neural Network, CDNN – Convolutional Deep Neural Networ

**Supplementary Table 3**. Performance of 14 machine learning methods for predicting the EQ-5D-5L index in Scenario 2

| **Catboost** | | | | | | | | | **AdaBoost** | | | | | | | | |
| --- | --- | --- | --- | --- | --- | --- | --- | --- | --- | --- | --- | --- | --- | --- | --- | --- | --- |
| **Metric** | **RMSE** | **MAE** | **R2** | **F** | **RCA** | **RCB** | **MTAE** | **G** | **Metric** | **RMSE** | **MAE** | **R2** | **F** | **RCA** | **RCB** | **MTAE** | **G** |
| **Min** | 0.162 | 0.087 | 0.459 | 0.876 | 0.967 | 0.991 | 0.060 | 0.942 | **Min** | 0.160 | 0.097 | 0.450 | 0.863 | 0.961 | 0.998 | 0.067 | 0.942 |
| **Max** | 0.162 | 0.087 | 0.465 | 0.894 | 0.967 | 0.991 | 0.060 | 0.945 | **Max** | 0.164 | 0.101 | 0.480 | 1.000 | 0.963 | 1.000 | 0.071 | 0.969 |
| **Mean** | 0.162 | 0.087 | 0.463 | 0.888 | 0.967 | 0.991 | 0.060 | 0.944 | **Mean** | 0.162 | 0.100 | 0.467 | 0.932 | 0.962 | 0.999 | 0.069 | 0.955 |
| **Median** | 0.162 | 0.087 | 0.463 | 0.890 | 0.967 | 0.991 | 0.060 | 0.944 | **Median** | 0.161 | 0.100 | 0.470 | 0.933 | 0.962 | 0.999 | 0.070 | 0.956 |
| **STD** | <0.001 | <0.001 | 0.002 | 0.005 | <0.001 | <0.001 | <0.001 | 0.001 | **STD** | 0.001 | 0.001 | 0.010 | 0.043 | 0.001 | 0.001 | 0.001 | 0.009 |
| **XGB** | | | | | | | | | **SVM** | | | | | | | | |
| **Metric** | **RMSE** | **MAE** | **R2** | **F** | **RCA** | **RCB** | **MTAE** | **G** | **Metric** | **RMSE** | **MAE** | **R2** | **F** | **RCA** | **RCB** | **MTAE** | **G** |
| **Min** | 0.164 | 0.088 | 0.386 | 0.889 | 0.963 | 0.991 | 0.061 | 0.944 | **Min** | 0.169 | 0.109 | 0.425 | 0.533 | 0.957 | 0.997 | 0.078 | 0.870 |
| **Max** | 0.174 | 0.094 | 0.444 | 0.940 | 0.966 | 0.994 | 0.068 | 0.954 | **Max** | 0.170 | 0.110 | 0.429 | 0.563 | 0.957 | 0.998 | 0.079 | 0.876 |
| **Mean** | 0.168 | 0.091 | 0.424 | 0.905 | 0.965 | 0.993 | 0.064 | 0.947 | **Mean** | 0.170 | 0.109 | 0.427 | 0.543 | 0.957 | 0.997 | 0.079 | 0.872 |
| **Median** | 0.166 | 0.090 | 0.436 | 0.897 | 0.965 | 0.993 | 0.064 | 0.946 | **Median** | 0.170 | 0.109 | 0.427 | 0.542 | 0.957 | 0.997 | 0.079 | 0.872 |
| **STD** | 0.004 | 0.002 | 0.023 | 0.018 | 0.001 | 0.001 | 0.002 | 0.004 | **STD** | <0.001 | <0.001 | 0.001 | 0.009 | <0.001 | <0.001 | <0.001 | 0.002 |
| **Decision Tree** | | | | | | | | | **LightGBM** | | | | | | | | |
| **Metric** | **RMSE** | **MAE** | **R2** | **F** | **RCA** | **RCB** | **MTAE** | **G** | **Metric** | **RMSE** | **MAE** | **R2** | **F** | **RCA** | **RCB** | **MTAE** | **G** |
| **Min** | 0.211 | 0.113 | 0.211 | 0.894 | 0.948 | 0.999 | 0.090 | 0.937 | **Min** | 0.163 | 0.090 | 0.462 | 0.732 | 0.966 | 0.990 | 0.061 | 0.912 |
| **Max** | 0.220 | 0.117 | 0.271 | 0.963 | 0.950 | 1.000 | 0.094 | 0.951 | **Max** | 0.163 | 0.090 | 0.467 | 0.746 | 0.966 | 0.991 | 0.062 | 0.915 |
| **Mean** | 0.216 | 0.114 | 0.237 | 0.928 | 0.950 | 0.999 | 0.091 | 0.945 | **Mean** | 0.163 | 0.090 | 0.465 | 0.740 | 0.966 | 0.991 | 0.062 | 0.914 |
| **Median** | 0.216 | 0.114 | 0.235 | 0.931 | 0.950 | 0.999 | 0.091 | 0.945 | **Median** | 0.163 | 0.090 | 0.465 | 0.739 | 0.966 | 0.991 | 0.062 | 0.913 |
| **STD** | 0.003 | 0.002 | 0.016 | 0.020 | 0.001 | <0.001 | 0.002 | 0.004 | **STD** | <0.001 | <0.001 | 0.001 | 0.005 | <0.001 | <0.001 | <0.001 | 0.001 |
| **KNN** | | | | | | | | | **SGD** | | | | | | | | |
| **Metric** | **RMSE** | **MAE** | **R2** | **F** | **RCA** | **RCB** | **MTAE** | **G** | **Metric** | **RMSE** | **MAE** | **R2** | **F** | **RCA** | **RCB** | **MTAE** | **G** |
| **Min** | 0.170 | 0.090 | 0.437 | 0.531 | 0.965 | 0.985 | 0.063 | 0.867 | **Min** | 0.166 | 0.097 | 0.428 | 0.561 | 0.961 | 0.992 | 0.068 | 0.876 |
| **Max** | 0.170 | 0.090 | 0.440 | 0.543 | 0.965 | 0.985 | 0.063 | 0.869 | **Max** | 0.168 | 0.099 | 0.439 | 0.682 | 0.963 | 0.994 | 0.070 | 0.901 |
| **Mean** | 0.170 | 0.090 | 0.439 | 0.539 | 0.965 | 0.985 | 0.063 | 0.869 | **Mean** | 0.167 | 0.098 | 0.432 | 0.649 | 0.962 | 0.993 | 0.069 | 0.894 |
| **Median** | 0.170 | 0.090 | 0.439 | 0.539 | 0.965 | 0.985 | 0.063 | 0.869 | **Median** | 0.167 | 0.098 | 0.432 | 0.659 | 0.962 | 0.993 | 0.070 | 0.896 |
| **STD** | <0.001 | <0.001 | 0.001 | 0.004 | <0.001 | <0.001 | <0.001 | 0.001 | **STD** | 0.001 | 0.001 | 0.003 | 0.036 | <0.001 | 0.001 | 0.001 | 0.007 |
| **Logistic Regression** | | | | | | | | | **LR** | | | | | | | | |
| **Metric** | **RMSE** | **MAE** | **R2** | **F** | **RCA** | **RCB** | **MTAE** | **G** | **Metric** | **RMSE** | **MAE** | **R2** | **F** | **RCA** | **RCB** | **MTAE** | **G** |
| **Min** | 0.203 | 0.097 | 0.415 | 0.094 | 0.956 | 0.958 | 0.078 | 0.752 | **Min** | 0.166 | 0.098 | 0.433 | 0.651 | 0.962 | 0.993 | 0.069 | 0.894 |
| **Max** | 0.207 | 0.098 | 0.432 | 0.118 | 0.957 | 0.959 | 0.079 | 0.758 | **Max** | 0.166 | 0.098 | 0.434 | 0.660 | 0.962 | 0.993 | 0.069 | 0.896 |
| **Mean** | 0.205 | 0.098 | 0.424 | 0.109 | 0.957 | 0.959 | 0.079 | 0.755 | **Mean** | 0.166 | 0.098 | 0.434 | 0.657 | 0.962 | 0.993 | 0.069 | 0.896 |
| **Median** | 0.204 | 0.098 | 0.425 | 0.111 | 0.957 | 0.959 | 0.079 | 0.756 | **Median** | 0.166 | 0.098 | 0.434 | 0.659 | 0.962 | 0.993 | 0.069 | 0.896 |
| **STD** | 0.001 | <0.001 | 0.006 | 0.008 | <0.001 | <0.001 | <0.001 | 0.002 | **STD** | <0.001 | <0.001 | <0.001 | 0.003 | <0.001 | <0.001 | <0.001 | 0.001 |
| **MLP** | | | | | | | | | **Passive Aggressive Regressor** | | | | | | | | |
| **Metric** | **RMSE** | **MAE** | **R2** | **F** | **RCA** | **RCB** | **MTAE** | **G** | **Metric** | **RMSE** | **MAE** | **R2** | **F** | **RCA** | **RCB** | **MTAE** | **G** |
| **Min** | 0.159 | 0.083 | 0.470 | 0.832 | 0.966 | 0.985 | 0.059 | 0.929 | **Min** | 0.176 | 0.105 | 0.153 | 0.355 | 0.888 | 0.905 | 0.076 | 0.725 |
| **Max** | 0.161 | 0.090 | 0.484 | 0.933 | 0.967 | 0.994 | 0.062 | 0.953 | **Max** | 0.289 | 0.236 | 0.380 | 0.883 | 0.958 | 0.998 | 0.203 | 0.927 |
| **Mean** | 0.160 | 0.087 | 0.477 | 0.898 | 0.966 | 0.991 | 0.061 | 0.946 | **Mean** | 0.214 | 0.151 | 0.315 | 0.606 | 0.934 | 0.959 | 0.120 | 0.838 |
| **Median** | 0.160 | 0.088 | 0.476 | 0.913 | 0.966 | 0.992 | 0.061 | 0.950 | **Median** | 0.208 | 0.138 | 0.331 | 0.649 | 0.941 | 0.959 | 0.107 | 0.850 |
| **STD** | 0.001 | 0.003 | 0.005 | 0.034 | 0.001 | 0.003 | 0.001 | 0.008 | **STD** | 0.038 | 0.042 | 0.070 | 0.171 | 0.022 | 0.034 | 0.041 | 0.064 |
| **DNN** | | | | | | | | | **CDNN** | | | | | | | | |
| **Metric** | **RMSE** | **MAE** | **R2** | **F** | **RCA** | **RCB** | **MTAE** | **G** | **Metric** | **RMSE** | **MAE** | **R2** | **F** | **RCA** | **RCB** | **MTAE** | **G** |
| **Min** | 0.160 | 0.085 | 0.423 | 0.718 | 0.963 | 0.989 | 0.060 | 0.908 | **Min** | 0.166 | 0.106 | 0.385 | 0.367 | 0.939 | 0.975 | 0.075 | 0.815 |
| **Max** | 0.167 | 0.096 | 0.470 | 0.935 | 0.967 | 0.995 | 0.068 | 0.954 | **Max** | 0.187 | 0.142 | 0.459 | 0.617 | 0.959 | 0.998 | 0.110 | 0.885 |
| **Mean** | 0.164 | 0.090 | 0.446 | 0.865 | 0.965 | 0.992 | 0.063 | 0.939 | **Mean** | 0.179 | 0.123 | 0.418 | 0.493 | 0.949 | 0.988 | 0.092 | 0.849 |
| **Median** | 0.163 | 0.088 | 0.450 | 0.903 | 0.966 | 0.992 | 0.062 | 0.946 | **Median** | 0.178 | 0.122 | 0.421 | 0.512 | 0.950 | 0.988 | 0.090 | 0.854 |
| **STD** | 0.003 | 0.004 | 0.018 | 0.083 | 0.002 | 0.002 | 0.003 | 0.017 | **STD** | 0.007 | 0.013 | 0.020 | 0.093 | 0.007 | 0.008 | 0.013 | 0.029 |

Abbreviations: AdaBoost - Adaptive Boosting, RMSE – root mean square error, MAE – mean absolute error, F – fairness metric, RCA - relative clinical accuracy, RCB - relative clinical bias, MTAE – mean truncated absolute error, G – goodness metric, STD – standard deviation, CatBoost - Categorical Boosting, AdaBoost - Adaptive Boosting, XGB - eXtreme Gradient Boosting regression, SVM - Support Vector Machine, LightGBM - Light Gradient Boosting Machine, KNN - K-Nearest Neighbours, SGD - Stochastic Gradient Descent, LR - Linear Regression, MLP - Multilayer Perceptron, DNN - Deep Neural Network, CDNN - Convolutional Deep Neural Network.

**Supplementary Table 4**. Performance of 14 machine learning methods for predicting the EQ-5D-5L index in Scenario 4

| **Catboost** | | | | | | | | | **AdaBoost** | | | | | | | | | |
| --- | --- | --- | --- | --- | --- | --- | --- | --- | --- | --- | --- | --- | --- | --- | --- | --- | --- | --- |
| **Metric** | **RMSE** | **MAE** | **R2** | **F** | **RCA** | **RCB** | **MTAE** | **G** | **Metric** | **RMSE** | **MAE** | **R2** | **F** | **RCA** | **RCB** | **MTAE** | **G** |  |
| **Min** | 0.158 | 0.085 | 0.436 | 0.470 | 0.968 | 0.994 | 0.059 | 0.863 | **Min** | 0.159 | 0.099 | 0.429 | 0.332 | 0.961 | 0.996 | 0.069 | 0.833 |  |
| **Max** | 0.158 | 0.085 | 0.437 | 0.473 | 0.968 | 0.994 | 0.059 | 0.864 | **Max** | 0.161 | 0.101 | 0.440 | 0.501 | 0.962 | 0.997 | 0.071 | 0.867 |  |
| **Mean** | 0.158 | 0.085 | 0.436 | 0.471 | 0.968 | 0.994 | 0.059 | 0.864 | **Mean** | 0.160 | 0.100 | 0.434 | 0.454 | 0.961 | 0.996 | 0.070 | 0.857 |  |
| **Median** | 0.158 | 0.085 | 0.436 | 0.470 | 0.968 | 0.994 | 0.059 | 0.864 | **Median** | 0.160 | 0.100 | 0.433 | 0.493 | 0.962 | 0.997 | 0.070 | 0.866 |  |
| **STD** | <0.001 | <0.001 | 0.001 | 0.001 | <0.001 | <0.001 | <0.001 | <0.001 | **STD** | 0.001 | 0.001 | 0.003 | 0.067 | 0.001 | <0.001 | 0.001 | 0.014 |  |
| **XGB** | | | | | | | | | **SVM** | | | | | | | | | |
| **Metric** | **RMSE** | **MAE** | **R2** | **F** | **RCA** | **RCB** | **MTAE** | **G** | **Metric** | **RMSE** | **MAE** | **R2** | **F** | **RCA** | **RCB** | **MTAE** | **G** |  |
| **Min** | 0.163 | 0.087 | 0.359 | 0.443 | 0.965 | 0.993 | 0.060 | 0.856 | **Min** | 0.170 | 0.107 | 0.361 | 0.373 | 0.958 | 0.995 | 0.077 | 0.837 |  |
| **Max** | 0.170 | 0.090 | 0.402 | 0.485 | 0.967 | 0.995 | 0.063 | 0.866 | **Max** | 0.170 | 0.107 | 0.363 | 0.376 | 0.958 | 0.995 | 0.077 | 0.838 |  |
| **Mean** | 0.165 | 0.088 | 0.392 | 0.467 | 0.966 | 0.994 | 0.061 | 0.862 | **Mean** | 0.170 | 0.107 | 0.362 | 0.374 | 0.958 | 0.995 | 0.077 | 0.837 |  |
| **Median** | 0.164 | 0.088 | 0.395 | 0.472 | 0.967 | 0.994 | 0.061 | 0.863 | **Median** | 0.170 | 0.107 | 0.362 | 0.374 | 0.958 | 0.995 | 0.077 | 0.837 |  |
| **STD** | 0.002 | 0.001 | 0.013 | 0.014 | 0.001 | 0.001 | 0.001 | 0.003 | **STD** | <0.001 | <0.001 | 0.001 | 0.001 | <0.001 | <0.001 | <0.001 | <0.001 |  |
| **Decision Tree** | | | | | | | | | **LightGBM** | | | | | | | | | |
| **Metric** | **RMSE** | **MAE** | **R2** | **F** | **RCA** | **RCB** | **MTAE** | **G** | **Metric** | **RMSE** | **MAE** | **R2** | **F** | **RCA** | **RCB** | **MTAE** | **G** |  |
| **Min** | 0.160 | 0.086 | 0.414 | 0.438 | 0.968 | 0.992 | 0.058 | 0.856 | **Min** | 0.168 | 0.091 | 0.352 | 0.424 | 0.966 | 0.993 | 0.062 | 0.852 |  |
| **Max** | 0.161 | 0.086 | 0.419 | 0.447 | 0.968 | 0.993 | 0.059 | 0.858 | **Max** | 0.169 | 0.091 | 0.359 | 0.430 | 0.966 | 0.993 | 0.062 | 0.853 |  |
| **Mean** | 0.160 | 0.086 | 0.417 | 0.444 | 0.968 | 0.993 | 0.058 | 0.857 | **Mean** | 0.169 | 0.091 | 0.356 | 0.427 | 0.966 | 0.993 | 0.062 | 0.852 |  |
| **Median** | 0.160 | 0.086 | 0.416 | 0.446 | 0.968 | 0.992 | 0.059 | 0.858 | **Median** | 0.168 | 0.091 | 0.356 | 0.427 | 0.966 | 0.993 | 0.062 | 0.852 |  |
| **STD** | <0.001 | <0.001 | 0.003 | 0.004 | <0.001 | <0.001 | <0.001 | 0.001 | **STD** | <0.001 | <0.001 | 0.002 | 0.002 | <0.001 | <0.001 | <0.001 | <0.001 |  |
| **KNN** | | | | | | | | | **SGD** | | | | | | | | | |
| **Metric** | **RMSE** | **MAE** | **R2** | **F** | **RCA** | **RCB** | **MTAE** | **G** | **Metric** | **RMSE** | **MAE** | **R2** | **F** | **RCA** | **RCB** | **MTAE** | **G** |  |
| **Min** | 0.192 | 0.102 | 0.264 | 0.578 | 0.957 | 0.995 | 0.079 | 0.877 | **Min** | 0.160 | 0.093 | 0.414 | 0.397 | 0.963 | 0.993 | 0.064 | 0.846 |  |
| **Max** | 0.192 | 0.102 | 0.265 | 0.583 | 0.957 | 0.995 | 0.079 | 0.878 | **Max** | 0.162 | 0.095 | 0.422 | 0.466 | 0.965 | 0.996 | 0.067 | 0.860 |  |
| **Mean** | 0.192 | 0.102 | 0.265 | 0.581 | 0.957 | 0.995 | 0.079 | 0.878 | **Mean** | 0.160 | 0.094 | 0.418 | 0.443 | 0.964 | 0.995 | 0.066 | 0.856 |  |
| **Median** | 0.192 | 0.102 | 0.265 | 0.581 | 0.957 | 0.995 | 0.079 | 0.878 | **Median** | 0.160 | 0.094 | 0.418 | 0.447 | 0.964 | 0.995 | 0.066 | 0.857 |  |
| **STD** | <0.001 | <0.001 | 0.001 | 0.002 | <0.001 | <0.001 | <0.001 | <0.001 | **STD** | 0.001 | 0.001 | 0.003 | 0.020 | 0.001 | 0.001 | 0.001 | 0.004 |  |
| **Logistic Regression** | | | | | | | | | **LR** | | | | | | | | | |
| **Metric** | **RMSE** | **MAE** | **R2** | **F** | **RCA** | **RCB** | **MTAE** | **G** | **Metric** | **RMSE** | **MAE** | **R2** | **F** | **RCA** | **RCB** | **MTAE** | **G** |  |
| **Min** | 0.182 | 0.088 | 0.332 | 0.145 | 0.962 | 0.971 | 0.069 | 0.776 | **Min** | 0.160 | 0.094 | 0.419 | 0.437 | 0.964 | 0.995 | 0.065 | 0.854 |  |
| **Max** | 0.184 | 0.088 | 0.347 | 0.176 | 0.962 | 0.973 | 0.070 | 0.784 | **Max** | 0.160 | 0.094 | 0.419 | 0.439 | 0.964 | 0.995 | 0.065 | 0.855 |  |
| **Mean** | 0.183 | 0.088 | 0.342 | 0.160 | 0.962 | 0.972 | 0.069 | 0.780 | **Mean** | 0.160 | 0.094 | 0.419 | 0.438 | 0.964 | 0.995 | 0.065 | 0.855 |  |
| **Median** | 0.183 | 0.088 | 0.343 | 0.160 | 0.962 | 0.972 | 0.069 | 0.780 | **Median** | 0.160 | 0.094 | 0.419 | 0.438 | 0.964 | 0.995 | 0.065 | 0.855 |  |
| **STD** | 0.001 | <0.001 | 0.005 | 0.009 | <0.001 | 0.001 | <0.001 | 0.002 | **STD** | <0.001 | <0.001 | <0.001 | <0.001 | <0.001 | <0.001 | <0.001 | <0.001 |  |
| **MLP** | | | | | | | | | **Passive Aggressive Regressor** | | | | | | | | | |
| **Metric** | **RMSE** | **MAE** | **R2** | **F** | **RCA** | **RCB** | **MTAE** | **G** | **Metric** | **RMSE** | **MAE** | **R2** | **F** | **RCA** | **RCB** | **MTAE** | **G** |  |
| **Min** | 0.176 | 0.095 | 0.305 | 0.501 | 0.957 | 0.990 | 0.068 | 0.863 | **Min** | 0.171 | 0.109 | 0.151 | 0.291 | 0.801 | 0.801 | 0.081 | 0.596 |  |
| **Max** | 0.186 | 0.107 | 0.353 | 0.651 | 0.963 | 0.999 | 0.078 | 0.894 | **Max** | 0.438 | 0.394 | 0.383 | 0.636 | 0.955 | 0.998 | 0.361 | 0.875 |  |
| **Mean** | 0.182 | 0.101 | 0.327 | 0.571 | 0.960 | 0.994 | 0.073 | 0.878 | **Mean** | 0.226 | 0.168 | 0.302 | 0.499 | 0.924 | 0.953 | 0.137 | 0.806 |  |
| **Median** | 0.183 | 0.101 | 0.325 | 0.570 | 0.959 | 0.994 | 0.074 | 0.878 | **Median** | 0.194 | 0.140 | 0.313 | 0.511 | 0.940 | 0.978 | 0.109 | 0.834 |  |
| **STD** | 0.003 | 0.004 | 0.015 | 0.054 | 0.002 | 0.002 | 0.003 | 0.011 | **STD** | 0.083 | 0.086 | 0.073 | 0.106 | 0.047 | 0.063 | 0.085 | 0.091 |  |
| **DNN** | | | | | | | | | **CDNN** | | | | | | | | | |
| **Metric** | **RMSE** | **MAE** | **R2** | **F** | **RCA** | **RCB** | **MTAE** | **G** | **Metric** | **RMSE** | **MAE** | **R2** | **F** | **RCA** | **RCB** | **MTAE** | **G** |  |
| **Min** | 0.160 | 0.086 | 0.337 | 0.391 | 0.965 | 0.990 | 0.060 | 0.843 | **Min** | 0.163 | 0.091 | 0.327 | 0.320 | 0.940 | 0.973 | 0.062 | 0.797 |  |
| **Max** | 0.175 | 0.091 | 0.421 | 0.489 | 0.967 | 0.996 | 0.063 | 0.865 | **Max** | 0.188 | 0.140 | 0.399 | 0.421 | 0.966 | 1.000 | 0.108 | 0.853 |  |
| **Mean** | 0.166 | 0.089 | 0.386 | 0.442 | 0.966 | 0.993 | 0.062 | 0.856 | **Mean** | 0.173 | 0.114 | 0.372 | 0.363 | 0.954 | 0.989 | 0.083 | 0.827 |  |
| **Median** | 0.166 | 0.089 | 0.396 | 0.441 | 0.966 | 0.993 | 0.062 | 0.856 | **Median** | 0.169 | 0.107 | 0.374 | 0.360 | 0.958 | 0.992 | 0.076 | 0.834 |  |
| **STD** | 0.005 | 0.001 | 0.029 | 0.033 | 0.001 | 0.002 | 0.001 | 0.008 | **STD** | 0.009 | 0.019 | 0.021 | 0.033 | 0.010 | 0.010 | 0.018 | 0.022 |  |

Abbreviations: AdaBoost - Adaptive Boosting, RMSE – root mean square error, MAE – mean absolute error, F – fairness metric, RCA - relative clinical accuracy, RCB - relative clinical bias, MTAE – mean truncated absolute error, G – goodness metric, STD – standard deviation, CatBoost - Categorical Boosting, AdaBoost - Adaptive Boosting, XGB - eXtreme Gradient Boosting regression, SVM - Support Vector Machine, LightGBM - Light Gradient Boosting Machine, KNN - K-Nearest Neighbours, SGD - Stochastic Gradient Descent, LR - Linear Regression, MLP - Multilayer Perceptron, DNN - Deep Neural Network, CDNN - Convolutional Deep Neural Network.

**Supplementary Table 5**. Performance of 14 machine learning methods for predicting the EQ-5D-5L index in Scenario 6

| **Catboost** | | | | | | | | | **AdaBoost** | | | | | | | | | |
| --- | --- | --- | --- | --- | --- | --- | --- | --- | --- | --- | --- | --- | --- | --- | --- | --- | --- | --- |
| **Metric** | **RMSE** | **MAE** | **R2** | **F** | **RCA** | **RCB** | **MTAE** | **G** | **Metric** | **RMSE** | **MAE** | **R2** | **F** | **RCA** | **RCB** | **MTAE** | **G** |  |
| **Min** | 0.158 | 0.086 | 0.436 | 0.473 | 0.967 | 0.994 | 0.059 | 0.864 | **Min** | 0.170 | 0.115 | 0.402 | 0.673 | 0.951 | 0.978 | 0.084 | 0.880 |  |
| **Max** | 0.158 | 0.086 | 0.438 | 0.476 | 0.967 | 0.994 | 0.059 | 0.865 | **Max** | 0.174 | 0.121 | 0.417 | 0.733 | 0.954 | 0.982 | 0.090 | 0.893 |  |
| **Mean** | 0.158 | 0.086 | 0.437 | 0.475 | 0.967 | 0.994 | 0.059 | 0.864 | **Mean** | 0.172 | 0.117 | 0.407 | 0.701 | 0.952 | 0.980 | 0.086 | 0.887 |  |
| **Median** | 0.158 | 0.086 | 0.437 | 0.475 | 0.967 | 0.994 | 0.059 | 0.864 | **Median** | 0.172 | 0.117 | 0.405 | 0.701 | 0.953 | 0.981 | 0.086 | 0.887 |  |
| **STD** | <0.001 | <0.001 | <0.001 | 0.001 | <0.001 | <0.001 | <0.001 | <0.001 | **STD** | 0.001 | 0.002 | 0.005 | 0.019 | 0.001 | 0.001 | 0.002 | 0.005 |  |
| **XGB** | | | | | | | | | **SVM** | | | | | | | | | |
| **Metric** | **RMSE** | **MAE** | **R2** | **F** | **RCA** | **RCB** | **MTAE** | **G** | **Metric** | **RMSE** | **MAE** | **R2** | **F** | **RCA** | **RCB** | **MTAE** | **G** |  |
| **Min** | 0.160 | 0.085 | 0.383 | 0.468 | 0.966 | 0.993 | 0.058 | 0.862 | **Min** | 0.169 | 0.107 | 0.367 | 0.349 | 0.958 | 0.995 | 0.076 | 0.833 |  |
| **Max** | 0.166 | 0.089 | 0.419 | 0.501 | 0.968 | 0.995 | 0.062 | 0.870 | **Max** | 0.169 | 0.107 | 0.369 | 0.352 | 0.958 | 0.995 | 0.077 | 0.833 |  |
| **Mean** | 0.163 | 0.087 | 0.399 | 0.483 | 0.967 | 0.994 | 0.061 | 0.865 | **Mean** | 0.169 | 0.107 | 0.369 | 0.351 | 0.958 | 0.995 | 0.076 | 0.833 |  |
| **Median** | 0.163 | 0.087 | 0.397 | 0.483 | 0.967 | 0.994 | 0.061 | 0.865 | **Median** | 0.169 | 0.107 | 0.369 | 0.350 | 0.958 | 0.995 | 0.076 | 0.833 |  |
| **STD** | 0.002 | 0.001 | 0.012 | 0.013 | 0.001 | 0.001 | 0.001 | 0.003 | **STD** | <0.001 | <0.001 | 0.001 | 0.001 | <0.001 | <0.001 | <0.001 | <0.001 |  |
| **Decision Tree** | | | | | | | | | **LightGBM** | | | | | | | | | |
| **Metric** | **RMSE** | **MAE** | **R2** | **F** | **RCA** | **RCB** | **MTAE** | **G** | **Metric** | **RMSE** | **MAE** | **R2** | **F** | **RCA** | **RCB** | **MTAE** | **G** |  |
| **Min** | 0.160 | 0.086 | 0.413 | 0.443 | 0.968 | 0.992 | 0.058 | 0.857 | **Min** | 0.167 | 0.091 | 0.359 | 0.431 | 0.965 | 0.993 | 0.063 | 0.853 |  |
| **Max** | 0.161 | 0.086 | 0.421 | 0.449 | 0.968 | 0.992 | 0.059 | 0.858 | **Max** | 0.168 | 0.092 | 0.364 | 0.436 | 0.966 | 0.993 | 0.063 | 0.854 |  |
| **Mean** | 0.160 | 0.086 | 0.419 | 0.445 | 0.968 | 0.992 | 0.058 | 0.857 | **Mean** | 0.168 | 0.091 | 0.361 | 0.434 | 0.965 | 0.993 | 0.063 | 0.854 |  |
| **Median** | 0.160 | 0.086 | 0.421 | 0.444 | 0.968 | 0.992 | 0.058 | 0.857 | **Median** | 0.168 | 0.091 | 0.361 | 0.434 | 0.965 | 0.993 | 0.063 | 0.854 |  |
| **STD** | <0.001 | <0.001 | 0.003 | 0.002 | <0.001 | <0.001 | <0.001 | <0.001 | **STD** | <0.001 | <0.001 | 0.001 | 0.002 | <0.001 | <0.001 | <0.001 | <0.001 |  |
| **KNN** | | | | | | | | | **SGD** | | | | | | | | | |
| **Metric** | **RMSE** | **MAE** | **R2** | **F** | **RCA** | **RCB** | **MTAE** | **G** | **Metric** | **RMSE** | **MAE** | **R2** | **F** | **RCA** | **RCB** | **MTAE** | **G** |  |
| **Min** | 0.176 | 0.095 | 0.331 | 0.563 | 0.961 | 0.994 | 0.071 | 0.877 | **Min** | 0.160 | 0.094 | 0.415 | 0.441 | 0.963 | 0.994 | 0.065 | 0.855 |  |
| **Max** | 0.176 | 0.095 | 0.332 | 0.566 | 0.961 | 0.994 | 0.071 | 0.878 | **Max** | 0.161 | 0.095 | 0.420 | 0.467 | 0.964 | 0.996 | 0.067 | 0.860 |  |
| **Mean** | 0.176 | 0.095 | 0.331 | 0.564 | 0.961 | 0.994 | 0.071 | 0.877 | **Mean** | 0.160 | 0.095 | 0.418 | 0.456 | 0.963 | 0.995 | 0.066 | 0.858 |  |
| **Median** | 0.176 | 0.095 | 0.331 | 0.564 | 0.961 | 0.994 | 0.071 | 0.877 | **Median** | 0.160 | 0.095 | 0.418 | 0.459 | 0.963 | 0.995 | 0.066 | 0.859 |  |
| **STD** | <0.001 | <0.001 | <0.001 | 0.001 | <0.001 | <0.001 | <0.001 | <0.001 | **STD** | <0.001 | 0.001 | 0.001 | 0.009 | <0.001 | 0.001 | 0.001 | 0.002 |  |
| **Logistic Regression** | | | | | | | | | **LR** | | | | | | | | | |
| **Metric** | **RMSE** | **MAE** | **R2** | **F** | **RCA** | **RCB** | **MTAE** | **G** | **Metric** | **RMSE** | **MAE** | **R2** | **F** | **RCA** | **RCB** | **MTAE** | **G** |  |
| **Min** | 0.180 | 0.087 | 0.347 | 0.148 | 0.962 | 0.971 | 0.068 | 0.777 | **Min** | 0.160 | 0.094 | 0.419 | 0.440 | 0.964 | 0.995 | 0.065 | 0.855 |  |
| **Max** | 0.182 | 0.088 | 0.362 | 0.190 | 0.962 | 0.974 | 0.070 | 0.787 | **Max** | 0.160 | 0.094 | 0.419 | 0.441 | 0.964 | 0.995 | 0.065 | 0.855 |  |
| **Mean** | 0.181 | 0.088 | 0.353 | 0.162 | 0.962 | 0.972 | 0.069 | 0.781 | **Mean** | 0.160 | 0.094 | 0.419 | 0.441 | 0.964 | 0.995 | 0.065 | 0.855 |  |
| **Median** | 0.181 | 0.088 | 0.352 | 0.160 | 0.962 | 0.972 | 0.069 | 0.780 | **Median** | 0.160 | 0.094 | 0.419 | 0.441 | 0.964 | 0.995 | 0.065 | 0.855 |  |
| **STD** | 0.001 | <0.001 | 0.004 | 0.011 | <0.001 | 0.001 | <0.001 | 0.003 | **STD** | <0.001 | <0.001 | <0.001 | <0.001 | <0.001 | <0.001 | <0.001 | <0.001 |  |
| **MLP** | | | | | | | | | **Passive Aggressive Regressor** | | | | | | | | | |
| **Metric** | **RMSE** | **MAE** | **R2** | **F** | **RCA** | **RCB** | **MTAE** | **G** | **Metric** | **RMSE** | **MAE** | **R2** | **F** | **RCA** | **RCB** | **MTAE** | **G** |  |
| **Min** | 0.176 | 0.095 | 0.270 | 0.488 | 0.955 | 0.987 | 0.069 | 0.856 | **Min** | 0.173 | 0.105 | 0.191 | 0.386 | 0.715 | 0.721 | 0.077 | 0.507 |  |
| **Max** | 0.196 | 0.109 | 0.368 | 0.680 | 0.962 | 1.000 | 0.081 | 0.897 | **Max** | 0.575 | 0.550 | 0.391 | 0.621 | 0.958 | 1.000 | 0.517 | 0.874 |  |
| **Mean** | 0.184 | 0.102 | 0.324 | 0.578 | 0.959 | 0.996 | 0.075 | 0.879 | **Mean** | 0.226 | 0.170 | 0.337 | 0.505 | 0.923 | 0.952 | 0.139 | 0.809 |  |
| **Median** | 0.182 | 0.103 | 0.328 | 0.575 | 0.959 | 0.997 | 0.075 | 0.881 | **Median** | 0.181 | 0.125 | 0.344 | 0.492 | 0.948 | 0.973 | 0.095 | 0.832 |  |
| **STD** | 0.006 | 0.004 | 0.027 | 0.061 | 0.002 | 0.004 | 0.004 | 0.013 | **STD** | 0.124 | 0.136 | 0.056 | 0.085 | 0.074 | 0.082 | 0.135 | 0.107 |  |
| **DNN** | | | | | | | | | **CDNN** | | | | | | | | | |
| **Metric** | **RMSE** | **MAE** | **R2** | **F** | **RCA** | **RCB** | **MTAE** | **G** | **Metric** | **RMSE** | **MAE** | **R2** | **F** | **RCA** | **RCB** | **MTAE** | **G** |  |
| **Min** | 0.161 | 0.085 | 0.318 | 0.420 | 0.963 | 0.990 | 0.060 | 0.850 | **Min** | 0.163 | 0.095 | 0.341 | 0.348 | 0.944 | 0.978 | 0.066 | 0.810 |  |
| **Max** | 0.181 | 0.095 | 0.417 | 0.506 | 0.967 | 0.998 | 0.067 | 0.871 | **Max** | 0.183 | 0.133 | 0.404 | 0.452 | 0.964 | 1.000 | 0.101 | 0.859 |  |
| **Mean** | 0.171 | 0.091 | 0.365 | 0.469 | 0.965 | 0.996 | 0.064 | 0.862 | **Mean** | 0.173 | 0.115 | 0.370 | 0.389 | 0.954 | 0.989 | 0.084 | 0.832 |  |
| **Median** | 0.172 | 0.092 | 0.358 | 0.476 | 0.965 | 0.996 | 0.064 | 0.864 | **Median** | 0.170 | 0.113 | 0.368 | 0.394 | 0.955 | 0.989 | 0.081 | 0.837 |  |
| **STD** | 0.006 | 0.003 | 0.030 | 0.032 | 0.001 | 0.002 | 0.002 | 0.007 | **STD** | 0.008 | 0.016 | 0.021 | 0.033 | 0.009 | 0.009 | 0.016 | 0.019 |  |

Abbreviations: AdaBoost - Adaptive Boosting, RMSE – root mean square error, MAE – mean absolute error, F – fairness metric, RCA - relative clinical accuracy, RCB - relative clinical bias, MTAE – mean truncated absolute error, G – goodness metric, STD – standard deviation, CatBoost - Categorical Boosting, AdaBoost - Adaptive Boosting, XGB - eXtreme Gradient Boosting regression, SVM - Support Vector Machine, LightGBM - Light Gradient Boosting Machine, KNN - K-Nearest Neighbours, SGD - Stochastic Gradient Descent, LR - Linear Regression, MLP - Multilayer Perceptron, DNN - Deep Neural Network, CDNN - Convolutional Deep Neural Network.

**Supplementary Table 6**. Performance of 14 machine learning methods for predicting the EQ-5D-5L index in Scenario 8

| **Catboost** | | | | | | | | | **AdaBoost** | | | | | | | | | |
| --- | --- | --- | --- | --- | --- | --- | --- | --- | --- | --- | --- | --- | --- | --- | --- | --- | --- | --- |
| **Metric** | **RMSE** | **MAE** | **R2** | **F** | **RCA** | **RCB** | **MTAE** | **G** | **Metric** | **RMSE** | **MAE** | **R2** | **F** | **RCA** | **RCB** | **MTAE** | **G** |  |
| **Min** | 1.58E-01 | 8.55E-02 | 4.36E-01 | 4.74E-01 | 9.67E-01 | 9.94E-01 | 5.90E-02 | 8.64E-01 | **Min** | 1.73E-01 | 1.19E-01 | 3.89E-01 | 7.31E-01 | 9.50E-01 | 9.77E-01 | 8.76E-02 | 8.89E-01 |  |
| **Max** | 1.58E-01 | 8.57E-02 | 4.38E-01 | 4.76E-01 | 9.67E-01 | 9.94E-01 | 5.91E-02 | 8.65E-01 | **Max** | 1.77E-01 | 1.22E-01 | 4.03E-01 | 7.48E-01 | 9.52E-01 | 9.79E-01 | 9.08E-02 | 8.94E-01 |  |
| **Mean** | 1.58E-01 | 8.56E-02 | 4.37E-01 | 4.75E-01 | 9.67E-01 | 9.94E-01 | 5.90E-02 | 8.64E-01 | **Mean** | 1.75E-01 | 1.20E-01 | 3.95E-01 | 7.40E-01 | 9.51E-01 | 9.78E-01 | 8.92E-02 | 8.92E-01 |  |
| **Median** | 1.58E-01 | 8.56E-02 | 4.37E-01 | 4.75E-01 | 9.67E-01 | 9.94E-01 | 5.90E-02 | 8.64E-01 | **Median** | 1.75E-01 | 1.20E-01 | 3.95E-01 | 7.41E-01 | 9.51E-01 | 9.78E-01 | 8.90E-02 | 8.92E-01 |  |
| **STD** | 5.99E-05 | 4.90E-05 | 4.09E-04 | 7.25E-04 | 2.37E-05 | 2.42E-05 | 4.31E-05 | 1.57E-04 | **STD** | 1.04E-03 | 9.94E-04 | 4.44E-03 | 5.68E-03 | 5.47E-04 | 6.97E-04 | 9.93E-04 | 1.40E-03 |  |
| **XGB** | | | | | | | | | **SVM** | | | | | | | | | |
| **Metric** | **RMSE** | **MAE** | **R2** | **F** | **RCA** | **RCB** | **MTAE** | **G** | **Metric** | **RMSE** | **MAE** | **R2** | **F** | **RCA** | **RCB** | **MTAE** | **G** |  |
| **Min** | 2.13E-01 | 1.26E-01 | 1.96E-01 | 8.07E-01 | 9.45E-01 | 9.81E-01 | 9.61E-02 | 9.06E-01 | **Min** | 1.70E-01 | 1.07E-01 | 3.59E-01 | 3.71E-01 | 9.57E-01 | 9.95E-01 | 7.70E-02 | 8.36E-01 |  |
| **Max** | 2.24E-01 | 1.30E-01 | 2.65E-01 | 8.53E-01 | 9.47E-01 | 9.82E-01 | 9.61E-02 | 9.13E-01 | **Max** | 1.71E-01 | 1.08E-01 | 3.61E-01 | 3.72E-01 | 9.58E-01 | 9.95E-01 | 7.70E-02 | 8.37E-01 |  |
| **Mean** | 2.19E-01 | 1.27E-01 | 2.27E-01 | 8.37E-01 | 9.46E-01 | 9.81E-01 | 9.78E-02 | 9.10E-01 | **Mean** | 1.70E-01 | 1.08E-01 | 3.61E-01 | 3.71E-01 | 9.58E-01 | 9.95E-01 | 7.71E-02 | 8.36E-01 |  |
| **Median** | 2.19E-01 | 1.27E-01 | 2.29E-01 | 8.37E-01 | 9.46E-01 | 9.81E-01 | 9.71E-02 | 9.10E-01 | **Median** | 1.70E-01 | 1.08E-01 | 3.61E-01 | 3.71E-01 | 9.58E-01 | 9.95E-01 | 7.71E-02 | 8.36E-01 |  |
| **STD** | 4.06E-03 | 1.46E-03 | 2.45E-02 | 1.47E-02 | 9.09E-04 | 4.45E-04 | 1.65E-03 | 2.95E-03 | **STD** | 1.06E-04 | 5.28E-05 | 7.10E-04 | 4.63E-04 | 2.82E-05 | 2.81E-05 | 5.11E-05 | 9.86E-05 |  |
| **Decision Tree** | | | | | | | | | **LightGBM** | | | | | | | | | |
| **Metric** | **RMSE** | **MAE** | **R2** | **F** | **RCA** | **RCB** | **MTAE** | **G** |  | **RMSE** | **MAE** | **R2** | **F** | **RCA** | **RCB** | **MTAE** | **G** |  |
| **Min** | 1.60E-01 | 8.60 E -02 | 4.13E-01 | 4.44E-01 | 9.68E-01 | 9.92E-01 | 5.83E-02 | 8.57E-01 | **Min** | 1.67E-01 | 9.12E-02 | 3.59E-01 | 4.30E-01 | 9.65E-01 | 9.93E-01 | 6.26 E -02 | 8.53E-01 |  |
| **Max** | 1.61E-01 | 8.62E-02 | 4.21E-01 | 4.49E-01 | 9.68E-01 | 9.92E-01 | 5.85E-02 | 8.58E-01 | **Max** | 1.68E-01 | 9.15E-02 | 3.64E-01 | 4.37E-01 | 9.65E-01 | 9.93E-01 | 6.29E-02 | 8.55E-01 |  |
| **Mean** | 1.60E-01 | 8.61E-02 | 4.18E-01 | 4.46E-01 | 9.68E-01 | 9.92E-01 | 5.84E-02 | 8.57E-01 | **Mean** | 1.68E-01 | 9.14E-02 | 3.61E-01 | 4.34E-01 | 9.65E-01 | 9.93E-01 | 6.28E-02 | 8.54E-01 |  |
| **Median** | 1.60E-01 | 8.60E-02 | 4.21E-01 | 4.44E-01 | 9.68E-01 | 9.92E-01 | 5.83E-02 | 8.57E-01 | **Median** | 1.68E-01 | 9.14E-02 | 3.61E-01 | 4.34E-01 | 9.65E-01 | 9.93E-01 | 6.28E-02 | 8.54E-01 |  |
| **STD** | 5.60E-04 | 1.11E-04 | 3.81E-03 | 2.34E-03 | 6.15E-05 | 6.21E-05 | 1.12E-04 | 4.68E-04 | **STD** | 2.19E-04 | 1.01E-04 | 1.62E-03 | 2.27E-03 | 4.61E-05 | 7.47E-05 | 8.36E-05 | 4.91E-04 |  |
| **KNN** | | | | | | | | | **SGD** | | | | | | | | | |
| **Metric** | **RMSE** | **MAE** | **R2** | **F** | **RCA** | **RCB** | **MTAE** | **G** |  | **RMSE** | **MAE** | **R2** | **F** | **RCA** | **RCB** | **MTAE** | **G** |  |
| **Min** | 1.92E-01 | 1.02E-01 | 2.66E-01 | 5.78E-01 | 9.56E-01 | 9.95E-01 | 7.91E-02 | 8.77E-01 | **Min** | 1.60E-01 | 9.20E-02 | 4.12E-01 | 4.01E-01 | 9.63E-01 | 9.92E-01 | 6.40E-02 | 8.46E-01 |  |
| **Max** | 1.93E-01 | 1.02E-01 | 2.68E-01 | 5.84E-01 | 9.56E-01 | 9.95E-01 | 7.93E-02 | 8.78E-01 | **Max** | 1.61E-01 | 9.55E-02 | 4.21E-01 | 4.67E-01 | 9.65E-01 | 9.96E-01 | 6.78E-02 | 8.61E-01 |  |
| **Mean** | 1.92E-01 | 1.02E-01 | 2.68E-01 | 5.81E-01 | 9.56E-01 | 9.95E-01 | 7.92E-02 | 8.78E-01 | **Mean** | 1.61E-01 | 9.39E-02 | 4.18E-01 | 4.41E-01 | 9.64E-01 | 9.94E-01 | 6.57E-02 | 8.55E-01 |  |
| **Median** | 1.92E-01 | 1.02E-01 | 2.68E-01 | 5.81E-01 | 9.56E-01 | 9.95E-01 | 7.92E-02 | 8.78E-01 | **Median** | 1.61E-01 | 9.38E-02 | 4.19E-01 | 4.44E-01 | 9.64E-01 | 9.95E-01 | 6.57E-02 | 8.56E-01 |  |
| **STD** | 2.24E-04 | 7.06E-05 | 8.60E-04 | 1.84E-03 | 3.54E-05 | 3.66E-05 | 6.42E-05 | 4.10E-04 | **STD** | 4.28E-04 | 1.06E-03 | 2.79E-03 | 2.09E-02 | 6.06E-04 | 1.22E-03 | 1.10E-03 | 4.63E-03 |  |
| **Logistic Regression** | | | | | | | | | **LR** | | | | | | | | | |
| **Metric** | **RMSE** | **MAE** | **R2** | **F** | **RCA** | **RCB** | **MTAE** | **G** |  | **RMSE** | **MAE** | **R2** | **F** | **RCA** | **RCB** | **MTAE** | **G** |  |
| **Min** | 1.78E-01 | 8.67E-02 | 3.47E-01 | 1.48E-01 | 9.62E-01 | 9.71E-01 | 6.78E-02 | 7.77E-01 | **Min** | 1.60E-01 | 9.35E-02 | 4.19E-01 | 4.40E-01 | 9.64E-01 | 9.95E-01 | 6.53E-02 | 8.55E-01 |  |
| **Max** | 1.82E-01 | 8.85E-02 | 3.68E-01 | 1.77E-01 | 9.63E-01 | 9.73E-01 | 6.97E-02 | 7.85E-01 | **Max** | 1.60E-01 | 9.36E-02 | 4.19E-01 | 4.42E-01 | 9.64E-01 | 9.95E-01 | 6.54E-02 | 8.55E-01 |  |
| **Mean** | 1.81E-01 | 8.77E-02 | 3.53E-01 | 1.61E-01 | 9.62E-01 | 9.72E-01 | 6.89E-02 | 7.80E-01 | **Mean** | 1.60E-01 | 9.36E-02 | 4.19E-01 | 4.41E-01 | 9.64E-01 | 9.95E-01 | 6.54E-02 | 8.55E-01 |  |
| **Median** | 1.81E-01 | 8.79E-02 | 3.52E-01 | 1.57E-01 | 9.62E-01 | 9.72E-01 | 6.91E-02 | 7.80E-01 | **Median** | 1.60E-01 | 9.36E-02 | 4.19E-01 | 4.41E-01 | 9.64E-01 | 9.95E-01 | 6.54E-02 | 8.55E-01 |  |
| **STD** | 9.94E-04 | 5.00E-04 | 5.82E-03 | 9.60E-03 | 2.85E-04 | 5.69E-04 | 5.18E-04 | 2.41E-03 | **STD** | 4.38E-06 | 2.07E-05 | 2.52E-05 | 3.55E-04 | 1.05E-05 | 2.09E-05 | 1.91E-05 | 7.80E-05 |  |
| **MLP** | | | | | | | | | **Passive Aggressive Regressor** | | | | | | | | | |
| **Metric** | **RMSE** | **MAE** | **R2** | **F** | **RCA** | **RCB** | **MTAE** | **G** |  | **RMSE** | **MAE** | **R2** | **F** | **RCA** | **RCB** | **MTAE** | **G** |  |
| **Min** | 1.81E-01 | 9.90E-02 | 2.59E-01 | 5.11E-01 | 9.55E-01 | 9.88E-01 | 7.25E-02 | 8.61E-01 | **Min** | 1.65E-01 | 9.72E-02 | 1.20E-01 | 3.63E-01 | 5.19E-01 | 5.22E-01 | 7.01E-02 | 2.89E-01 |  |
| **Max** | 1.99E-01 | 1.09E-01 | 3.44E-01 | 6.94E-01 | 9.60E-01 | 9.98E-01 | 8.15E-02 | 8.96E-01 | **Max** | 9.25E-01 | 9.05E-01 | 3.98E-01 | 6.05E-01 | 9.61E-01 | 9.94E-01 | 8.72E-01 | 8.70E-01 |  |
| **Mean** | 1.88E-01 | 1.04E-01 | 3.13E-01 | 6.07E-01 | 9.58E-01 | 9.94E-01 | 7.65E-02 | 8.83E-01 | **Mean** | 2.67E-01 | 2.13E-01 | 3.26E-01 | 4.84E-01 | 8.99E-01 | 9.27E-01 | 1.83E-01 | 7.78E-01 |  |
| **Median** | 1.88E-01 | 1.04E-01 | 3.23E-01 | 6.04E-01 | 9.58E-01 | 9.95E-01 | 7.64E-02 | 8.86E-01 | **Median** | 1.84E-01 | 1.24E-01 | 3.50E-01 | 4.66E-01 | 9.48E-01 | 9.88E-01 | 9.46E-02 | 8.44E-01 |  |
| **STD** | 5.71E-03 | 3.86E-03 | 3.00E-02 | 5.99E-02 | 1.82E-03 | 3.62E-03 | 3.30E-03 | 1.22E-02 | **STD** | 2.35E-01 | 2.49E-01 | 8.11E-02 | 9.94E-02 | 1.36E-01 | 1.47E-01 | 2.47E-01 | 1.77E-01 |  |
| **DNN** | | | | | | | | | **CDNN** | | | | | | | | | |
| **Metric** | **RMSE** | **MAE** | **R2** | **F** | **RCA** | **RCB** | **MTAE** | **G** | **Metric** | **RMSE** | **MAE** | **R2** | **F** | **RCA** | **RCB** | **MTAE** | **G** |  |
| **Min** | 1.62E-01 | 8.49E-02 | 3.37E-01 | 4.35E-01 | 9.63E-01 | 9.91E-01 | 5.92E-02 | 8.56E-01 | **Min** | 1.65E-01 | 9.60E-02 | 3.50E-01 | 3.31E-01 | 9.43E-01 | 9.78E-01 | 6.66E-02 | 8.06E-01 |  |
| **Max** | 1.76E-01 | 9.50E-02 | 4.12E-01 | 5.09E-01 | 9.67E-01 | 9.99E-01 | 6.64E-02 | 8.72E-01 | **Max** | 1.83E-01 | 1.35E-01 | 3.86E-01 | 4.75E-01 | 9.63E-01 | 9.99E-01 | 1.03E-01 | 8.65E-01 |  |
| **Mean** | 1.69E-01 | 8.97E-02 | 3.74E-01 | 4.74E-01 | 965E-01 | 9.95E-01 | 6.28E-02 | 8.63E-01 | **Mean** | 1.72E-01 | 1.11E-01 | 3.69E-01 | 3.97E-01 | 9.56E-01 | 9.92E-01 | 8.06E-02 | 8.37E-01 |  |
| **Median** | 1.68E-01 | 8.93E-02 | 3.75E-01 | 4.76E-01 | 9.66E-01 | 9.96E-01 | 6.23E-02 | 8.63E-01 | **Median** | 1.69E-01 | 1.05E-01 | 3.73E-01 | 3.99E-01 | 9.59E-01 | 9.95E-01 | 7.41E-02 | 8.45E-01 |  |
| **STD** | 4.85E-03 | 3.32E-03 | 2.45E-02 | 2.51E-02 | 1.23E-03 | 2.54E-03 | 2.23E-03 | 5.58E-03 | **STD** | 6.93E-03 | 1.54E-02 | 1.49E-02 | 5.36E-02 | 8.00E-03 | 8.59E-03 | 1.45E-02 | 2.26E-02 |  |

Abbreviations: AdaBoost - Adaptive Boosting, RMSE – root mean square error, MAE – mean absolute error, F – fairness metric, RCA - relative clinical accuracy, RCB - relative clinical bias, MTAE – mean truncated absolute error, G – goodness metric, STD – standard deviation, CatBoost - Categorical Boosting, AdaBoost - Adaptive Boosting, XGB - eXtreme Gradient Boosting regression, SVM - Support Vector Machine, LightGBM - Light Gradient Boosting Machine, KNN - K-Nearest Neighbours, SGD - Stochastic Gradient Descent, LR - Linear Regression, MLP - Multilayer Perceptron, DNN - Deep Neural Network, CDNN - Convolutional Deep Neural Network.

**Supplementary Table 7**. Description of the source data

| # | Short description | Time frame | N of participants with EQ-5D-5L data / survey method | Age of resp. (years) |
| --- | --- | --- | --- | --- |
| 1 | Production and validation of the Patient Activation Measure (PAM-13) for Hungary (1) | April 2020 | N=900; online survey | >=40 |
| 2 | Measurement of the health status and well-being of the Hungarian population (2-4) | During 2019 | N=2020; computer-assisted personal interviews | >=18 |
| 3 | Expectations of the public about future health and living conditions (5-6) | Early 2019 | N=1000; online survey | >=18 |
| 4 | eHealth literacy, shared decision making and patients’ experiences with healthcare - Questionnaire Survey of the Hungarian Population (7-12) | Early 2019 | N=1000; online survey | >=18 |
| 5 | Assessment of well-being: a survey of the general population (13) | May-June 2019 | N=1000; computer-assisted personal interviews | >=18 |
| 6 | Survey on the musculoskeletal health of the population (14) | May-June 2020 | N=2004; online survey | >=18 |
| 7 | Knowledge of and attitudes towards implantable medical devices among the general population (15) | July 2021 | N=1400; online survey | >=40 |

References:

1. Zrubka, Z. et al. Validation of the PAM-13 instrument in the Hungarian general population 40 years old and above. Eur J Health Econ. 23, 1341-1355 (2022).
2. Baji, P. et al. Capability of well-being: validation of the Hungarian version of the ICECAP-A and ICECAP-O questionnaires and population normative data. Quality of Life Research. 29, 2863-2874 (2020).
3. Baji, P. et al. Comparing the measurement properties of the ICECAP-A and ICECAP-O instruments in ages 50-70: a cross-sectional study on a representative sample of the Hungarian general population. Eur J Health Econ. 22, 1453-1466 (2021).
4. Péntek, M. et al. Musculoskeletal health and capability wellbeing: Associations between the HAQ-DI, ICECAP-A and ICECAP-O measures in a population survey. Musculoskelet Sci Pract. 55, 102420 (2021).
5. Péntek, M. et al. Subjective expectations regarding ageing: a cross-sectional online population survey in Hungary. Eur J Health Econ. 20, 17-30 (2019).
6. Zrubka, Z. et al. Comparing actuarial and subjective healthy life expectancy estimates: A cross-sectional survey among the general population in Hungary. PLoS One. 17, e0264708 (2022).
7. Zrubka, Z. et al. Psychometric properties of the Hungarian version of the eHealth Literacy Scale. Eur J Health Econ. 20, 57-69 (2019).
8. Brito Fernandes, Ó. et al. Patient experiences with outpatient care in Hungary: results of an online population survey. Eur J Health Econ. 20, 79-90 (2019).
9. Brito Fernandes, Ó. et al. Self-Reported Waiting Times for Outpatient Health Care Services in Hungary: Results of a Cross-Sectional Survey on a National Representative Sample. Int. J. Environ. Res. Public Health. 18 (2021).
10. Brito Fernandes, Ó. et al. Eliciting preferences for outpatient care experiences in Hungary: A discrete choice experiment with a national representative sample. PLoS One. 15, e0235165 (2020).
11. Lucevic, A. et al. Unmet medical needs in ambulatory care in Hungary: forgone visits and medications from a representative population survey. Eur J Health Econ. 20, 71-78 (2019).
12. Rencz, F. et al. Validity and reliability of the 9-item Shared Decision Making Questionnaire (SDM-Q-9) in a national survey in Hungary. Eur J Health Econ. 20, 43-55 (2019).
13. Zrubka, Z. et al. Exploring eHealth Literacy and Patient-Reported Experiences With Outpatient Care in the Hungarian General Adult Population: Cross-Sectional Study. J. Med. Internet Res. 22, e19013 (2020).
14. Farkas, M. et al. Development of Population Tariffs for the ICECAP-A Instrument for Hungary and their Comparison With the UK Tariffs. Value Health. 24, 1845-1852 (2021).
15. Holgyesi, A. et al. Validation of the Musculoskeletal Health Questionnaire in a general population sample: a cross-sectional online survey in Hungary. BMC Musculoskelet. Disord. 23, 771 (2022).
16. Holgyesi, A. et al. Epidemiology and patients' self-reported knowledge of implantable medical devices: Results of a cross-sectional survey in Hungary. PLoS One. 18, e0284577 (2023).

**Supplementary Table 8.** Standard questionnaires used in the study

| **EQ-5D-5L** |
| --- |
| The EQ-5D-5L questionnaire comprises 2 parts. The descriptive system asks about respondents’ problems in 5 health dimensions (mobility, self-care, usual activities, pain / discomfort and anxiety / depression). Problems are recorded in 5 levels (1-none, 2-mild, 3-moderate, 4-severe, 5-extreme), providing 3525 (55) discrete health states (1). Utilities were calculated by attaching the Hungarian general population’s preference weights to each health state (2). Hungarian EQ-5D-5L utilities range between 1 (no problems in any dimension) and –0.848 (extreme problems in all dimensions). The minimum clinically important difference (MCID) in the Hungarian EQ-5D-5L utility values is 0.066 (3). The second part, the EQ VAS assesses respondent’s current health on a visual analogue scale (thermometer), between 100 (best possible health) and 0 (worst possible health). |
| **Minimum European Health Module (MEHM)** |
| The MEHM evaluates the general health state of respondents with a set of three questions on self-perceived health (very good/good/fair/bad/very bad); (2) long-standing illness (yes/no) and (3) activity limitations due to health problems for more than 6 months measured with the Global Activity Limitation Indicator (GALI) (severely limited/limited but not severely or/not limited at all) (4). |

References:

1. Herdman M, Gudex C, Lloyd A, Janssen M, Kind P, Parkin D, et al. Development and preliminary testing of the new five-level version of EQ-5D (EQ-5D-5L). Qual Life Res. 2011;20(10):1727-36.
2. Rencz F, Brodszky V, Gulacsi L, Golicki D, Ruzsa G, Pickard AS, et al. Parallel Valuation of the EQ-5D-3L and EQ-5D-5L by Time Trade-Off in Hungary. Value Health. 2020;23(9):1235-45.
3. Zrubka Z, Hölgyesi Á, Neshat M, Nezhad HM, Mirjalili S, Kovács L, et al. Towards a single goodness metric of clinically relevant, accurate, fair and unbiased machine learning predictions of health-related quality of life. In: Szakál A, editor. 2023 IEEE 27th International Conference on Intelligent Engineering Systems (INES); Budapest, Hungary: IEEE Hungary Section; 2023. p. 000285-90.
4. Cox B, van Oyen H, Cambois E, Jagger C, le Roy S, Robine JM, et al. The reliability of the Minimum European Health Module. International journal of public health. 2009;54(2):55-60.

**Supplementary Table 9.** Overview of research scenarios

|  | | **Missing data handling strategies** | | | |
| --- | --- | --- | --- | --- | --- |
|  |  | **Deletion** | **Multiple Imputation by Chained Equations** | | |
|  |  |  | **No EQ-5D-5L index** | **EQ-5D-5L index in the train data** | **EQ-5D-5L index in the train and cross-validation data** |
| **Datasets** | **Demographic variables + MEHM^a^** | Scenario 2 | Scenario 4 | Scenario 6 | Scenario 8 |
|  | **Demographic variables^b^** | Scenario 1 | Scenario 3 | Scenario 5 | Scenario 7 |

^a^ All four scenarios were implemented and model performance was evaluated

^b^ Only one scenario was implemented without MEHM: the counterpart of the scenario featuring both demographic variables and MEHM with best prediction performance

Abbreviations: MEHM – Minimum European Health Module

**Supplementary Table** **10.** Technical settings of the machine learning methods

| # | Acronym | Full name | Hyper-parameters |
| --- | --- | --- | --- |
| 1 | KNN | K Nearest Neighbors | (K=Number of neighbours) |
| 2 | LoR | Logistic Regression | solver=’lbfgs’, penalty=’l2’,tol=0.0001, C=1.0, max- iter=100 |
| 3 | LR | Linear Regression | pre-defined settings (scikit-learn) |
| 4 | PAR | Passive Aggressive Regressor | C=1.0, fit intercept=True, max iter=1000, tol=0.001, early stopping=False, validation fraction=0.1, n iter no change=5, shuffle=True, verbose=0, loss=’epsilon insen- sitive’, epsilon=0.1 |
| 5 | SGD | Stochastic gradient descent | loss=’squared error’, penalty=’l2’, alpha=0.0001, l1 ra- tio=0.15, max iter=1000, tol=0.001, shuffle=True, ep- silon=0.1, learning rate=’invscaling’, eta0=0.01, power t=0.25, |
| 6 | SVM | Support Vecor Machine | kernel='rbf', degree=3, gamma='scale', coef0=0.0, tol=0.001, C=1.0, epsilon=0.1 |
| 7 | DT | Decision Tree Regressor | criterion=’squared error’, splitter=’best’, max depth= D, min samples split=2, min samples leaf=1, min weight fraction leaf=0.0, |
| 8 | MLP | Multilayer Perceptron | solver=’adam’, activation=’relu’, alpha=1e-4, hidden layer sizes=(200,20,), max iter=1000 |
| 9 | XGBoost | eXtreme Gradient Boosting | n_estimators=10, max_depth=10, gamma=2, eta=0.3, reg_alpha=0.5, reg_lambda=0.5, subsample=0.5 |
| 10 | AdaBoost | Adaptive Boosting | number estimators=50, learning rate=1.0, loss=’linear’, base estimator=’deprecated’ |
| 11 | CatBoost | Categorical Boosting | iterations=10000, learning_rate=0.01, depth=6, l2_leaf_reg=3, loss_function='RMSE' |
| 12 | LightGBM | Light Gradient Boosting | Metric= 'rmse', num_iterations=2000, num_leaves= 2000, learning_rate= 0.001, feature_fraction= 0.9, max_depth= 20 |
| 13 | DNN | Deep Neural Network | epochs=200, batch_size=32, loss='mean_squared_error', optimizer=Adam, lr = 1e-4 |
| 14 | CDNN | Convolutional Deep Neural Network | filters=32, 64, 128, kernel_size=3, activation='relu', pool_size=2, loss='mean_squared_error', optimizer= Adam, lr = 1e-4, epochs=1000, batch_size=32 |

**Supplementary Table 11**. Performance metrics

| Metrics | Definition | Equation |
| --- | --- | --- |
| MSE | mean square error | $\mathrm{MSE}=\frac{1}{N_{s}}\sum_{k=1}^{N_{s}} \left( f_{e}(k)-f_{t}(k) \right)^{2}$ |
| RMSE | root mean square error | $\text{RMSE }=\sqrt{\frac{1}{N_{s}}\sum_{k=1}^{N_{s}} \left( f_{e}(k)-f_{t}(k) \right)^{2}}$ |
| AE | absolute error | $\mathrm{AE}=\left\vert f_{e}(k)-f_{t}(k) \right\vert$ |
| MAE | mean absolute error | $\text{MAE }=\frac{1}{N_{s}}\sum_{k=1}^{N_{s}} \left\vert f_{e}(k)-f_{t}(k) \right\vert$ |
| TE | truncated error | $\mathrm{TE}=\left\{ \begin{aligned} &E-\frac{1}{2}MCID &&\text{ if }\left( E\geq\frac{1}{2}MCID \right) \\ &E+\frac{1}{2}MCID &&\text{ if }\left( E<-\frac{1}{2}MCID \right) \\ &0 &&\text{ if }\left. \left( E<\left\vert\frac{1}{2}MCID \right. \right) \right\vert\end{aligned} \right.$ |
| MTAE | mean truncated absolute error | $MTAE=\frac{1}{N_{g}}\sum_{k=1}^{N_{g}} \left\vert TE_{k} \right\vert$ |
| MAPE | maximum perceivable prediction error | $MAPE=1.848-\left( \frac{1}{2}MCID \right)$ |
| RCA | relative clinical accuracy | $RCA=\frac{(MAPE-MTAE)}{MAPE}$ |
| RCB | relative clinical bias | $RCB=\frac{\left( MAPE-\left\vert\frac{1}{N_{g}}\sum_{k=1}^{N_{g}} \left( TE_{k}\mid\right) \right. \right.}{MAPE}$ |
| R-value | pearson correlation coefficient | $R=\frac{\frac{1}{N_{s}}\sum_{k=1}^{N_{s}} \left( f_{e}(k)-\bar{f}_{e} \right)\left( f_{t}(k)-\bar{f}_{t} \right)}{\sqrt{\frac{1}{N_{s}}\sum_{k=1}^{N_{s}} \left( f_{e}(k)-\bar{f}_{e} \right)^{2}}\times\sqrt{\frac{1}{N_{s}}\sum_{k=1}^{N_{s}} \left( f_{e}(k)-\bar{f}_{t} \right)^{2}}}$ |
| F | fairness | $F=\prod_{i=1}^{m} \left( 1-PPS\left[ TE,P_{i} \right] \right)\times\left( 1-PPS\left( TAE,P_{i} \right) \right.$ |
| G | Goodness (hybrid metric) | $G=w\times(RCA\times RCB)+(1-w)\times F$ |

**Figures**

| 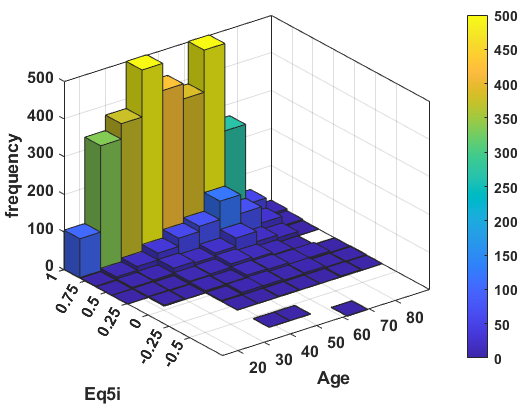 | 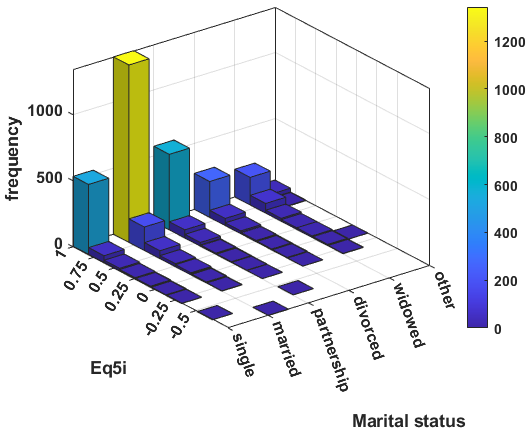 | 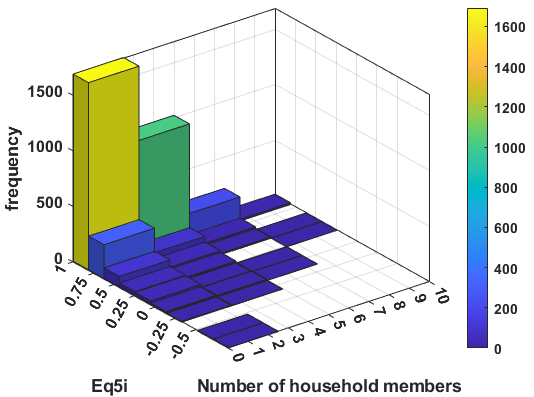 |
| --- | --- | --- |
| 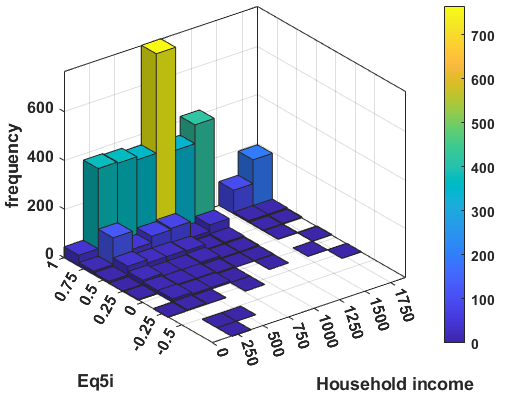 | 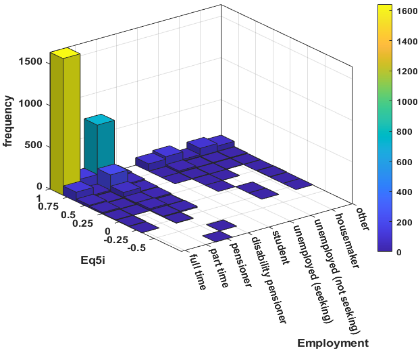 | 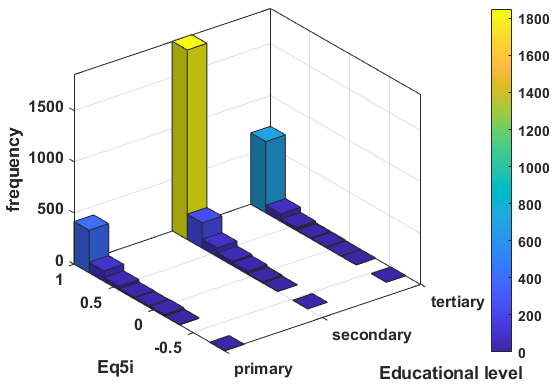 |
| 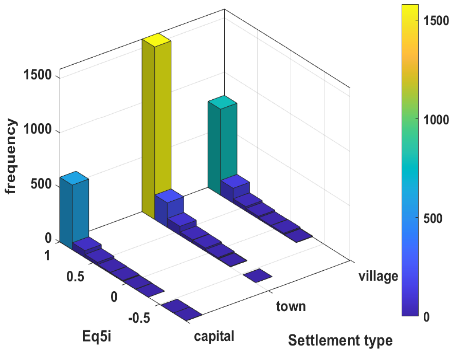 | 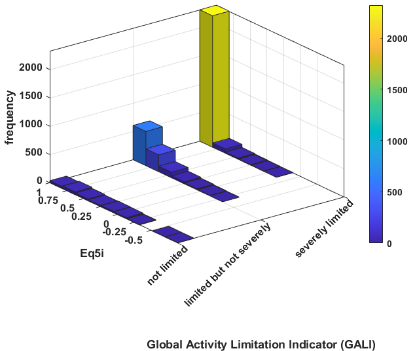 | 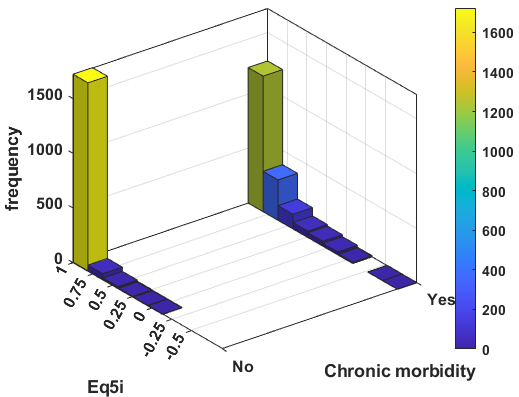 |

**Supplementary Figure 1.** Joint distribution of the EQ-5D-5L index with predictor variables (Scenario 2, training dataset)


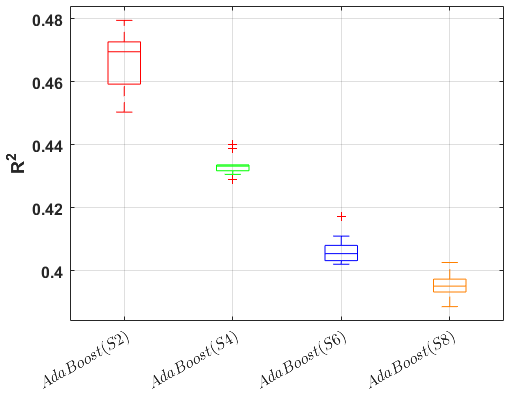


**Supplementary Figure 2*.*** The performance of AdaBoost across the four scenarios in terms of R^2^.

| 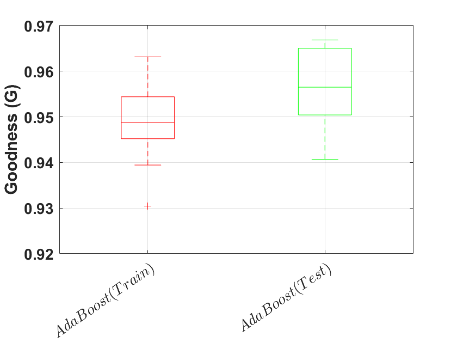 | 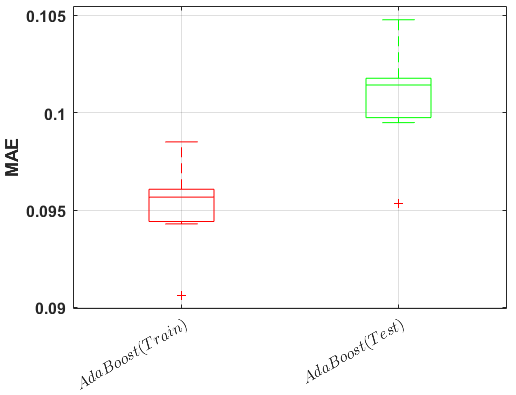 |
| --- | --- |
| (a) | (b) |
| 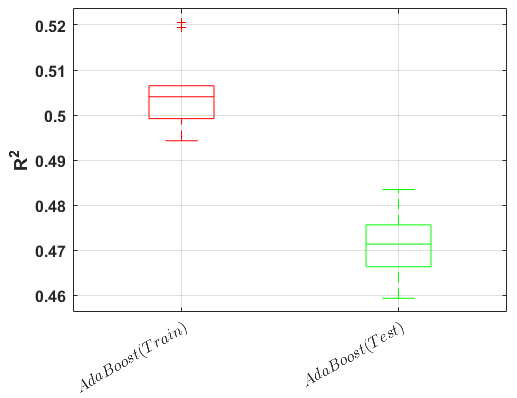 | 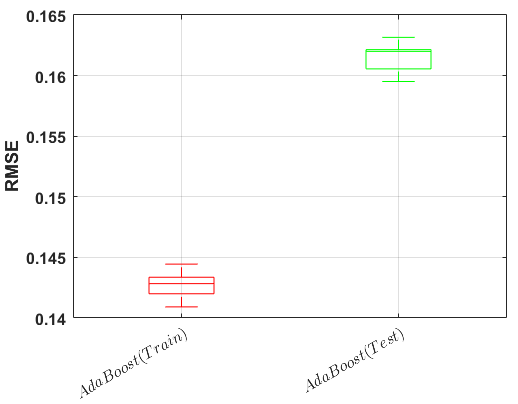 |
| (c) | (d) |

**Supplementary Figure 3.** The performance of AdaBoost based on scenario 2 compared for train and test dataset in terms of (a) G-score, (b) MAE, (c) R2, and (d) RMSE.

| 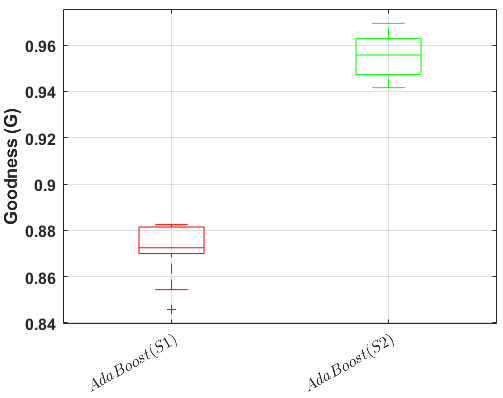 | 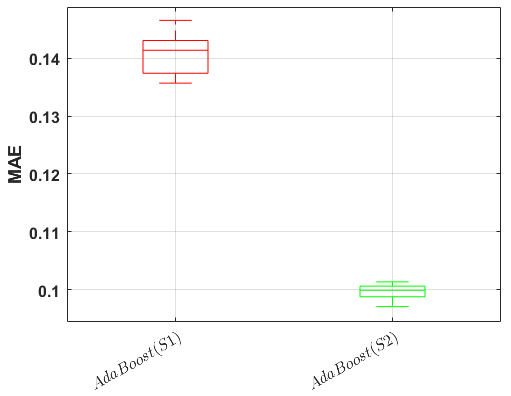 | 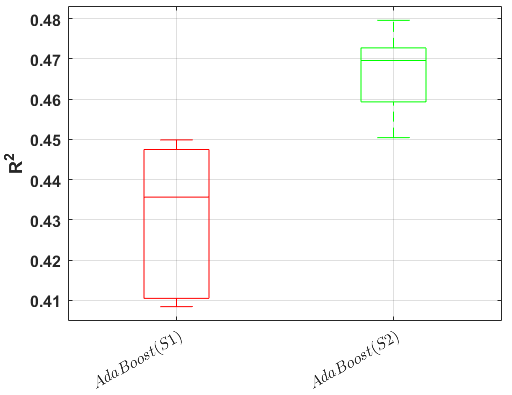 |
| --- | --- | --- |
| (a) | (b) | (c) |

Supplementary Figure 4. Comparison of AdaBoost’s prediction accuracy between Scenario 1 and 2 using a) the G score, b) the MAE, and c) the R^2^.


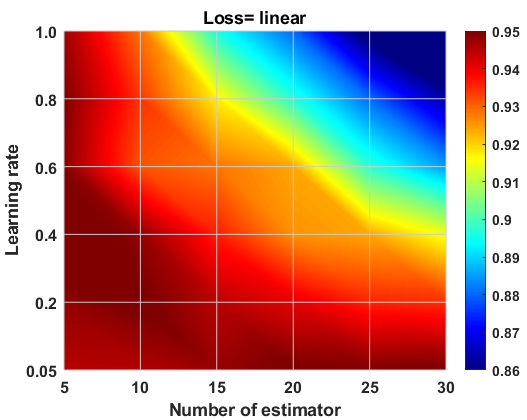


Supplementary Figure 5. Hyper-parameters tuning using the Grid search approach

| 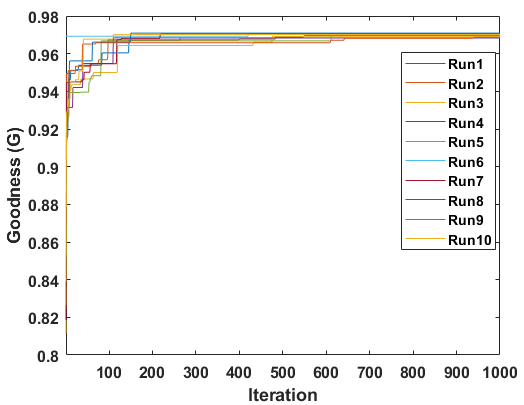 | 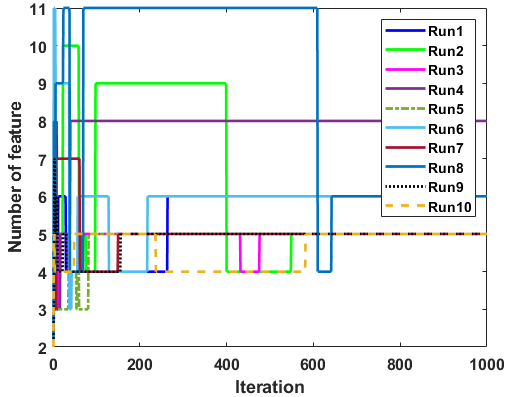 | 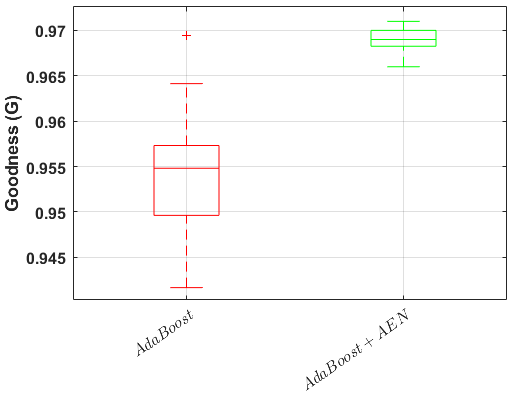 |
| --- | --- | --- |
| (a) | (b) | (c) |

**Supplementary Figure** **6.** A comparison and performance of hybrid feature selection method (AEN) and AdaBoost. (a) ten independent runs in terms of Goodness (G), (b) feature numbers variations during the optimisation process, (c) the impact of AEN on the AdaBoost performance


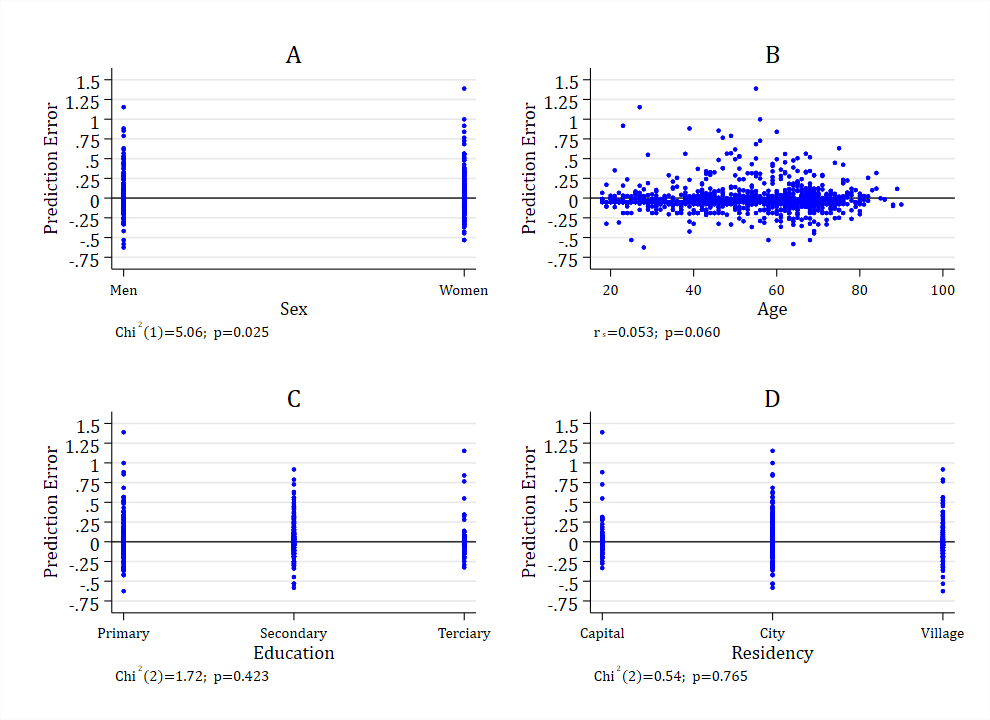


**Supplementary Figure 7.** Distribution of prediction error by (a) sex, (b) age, (c) education and (d) residency. P-values, chi-squared statistics, and degrees of freedom (in parentheses) are reported for binary (sex) and multi-group (education, residency) comparisons, conducted using the Mann–Whitney U test and Kruskal–Wallis test, respectively. The reported coefficient for age represents Spearman’s rank correlation coefficient.
